# Magnetic nanoplatforms at the tumor redox interface: from catalytic ROS amplification to ferroptosis and multimodal cancer therapy

**DOI:** 10.3389/fonc.2026.2001431

**Published:** 2026-10-20

**Authors:** Gulnara Kurmanalina, Vadim Medovchshikov, Setareh Valanik, Raisa Aringazina

**Affiliations:** 1 Department of Internal Diseases No. 2, West Kazakhstan Marat Ospanov Medical University, Aktobe, Kazakhstan; 2 Student Research Committee, School of Dentistry, Shiraz University of Medical Sciences, Shiraz, Iran; 3 Department of Internal Diseases No. 1, West Kazakhstan Marat Ospanov Medical University, Aktobe, Kazakhstan

**Keywords:** chemodynamic therapy, ferroptosis, magnetic nanoparticles, reactive oxygen species (ROS), tumor microenvironment

## Abstract

Reactive oxygen species (ROS)-mediated cancer therapy has emerged as a promising strategy for inducing oxidative stress and cancer cell death. Among available nanomaterials, magnetic nanoparticles (MNPs) are particularly attractive because of their magnetic responsiveness, tunable physicochemical properties, and capacity to generate or amplify intracellular ROS within the tumor microenvironment. MNPs can disrupt cellular redox homeostasis through Fenton and Fenton-like reactions, chemodynamic therapy, ferroptosis, and mitochondrial dysfunction, thereby activating multiple cancer cell death pathways. Recent advances in nanomaterial engineering have further improved their therapeutic potential through surface functionalization, compositional optimization, targeted delivery, and stimuli-responsive design. ROS-generating magnetic nanoplatforms have also been integrated with chemotherapy, magnetic hyperthermia, photothermal therapy, photodynamic therapy, and immunotherapy to achieve synergistic antitumor effects. However, important challenges remain, including limited ROS-generation efficiency, tumor heterogeneity, biosafety concerns, and barriers to clinical translation. This review summarizes the fundamental principles and mechanisms of ROS-mediated cancer therapy using MNPs, discusses major engineering and combination-therapy strategies, and evaluates current limitations and future perspectives. Particular emphasis is placed on relationships between nanoparticle composition, structure, catalytic behavior, tumor responsiveness, and therapeutic outcomes and clinical translation strategies. These insights may support the rational design of next-generation magnetic nanoplatforms for more effective and precise cancer treatment.

## 1 Introduction

Cancer remains a major global health burden and continues to challenge modern medicine despite substantial progress in diagnosis and treatment. According to the latest global estimates, there were close to 20 million new cancer cases and 9.7 million cancer deaths worldwide in 2022, and the burden is projected to rise further in the coming decades (1). Conventional modalities such as surgery, chemotherapy, radiotherapy, and immunotherapy have improved survival for many patients, yet their effectiveness is often limited by systemic toxicity, incomplete tumor selectivity, relapse, and the adaptive nature of the tumor microenvironment. These limitations have encouraged the development of nanomedicine-based strategies designed to improve tumor accumulation, therapeutic precision, and multimodal treatment performance (2, 3).

Within this context, reactive oxygen species (ROS) have emerged as a particularly attractive therapeutic target. ROS are biologically active molecules that at low to moderate levels play a dual role in cancer (4). They are involved in signaling pathways that support proliferation, invasion, and survival, while excessive accumulation of ROS overwhelms antioxidant defenses and causes oxidative damage. In cancer cells, this redox balance is often already strained, which makes malignant cells more vulnerable to further ROS elevation than normal tissues (5). As a result, strategies that deliberately amplify ROS beyond the tolerance threshold of cancer cells have gained considerable attention as a route to selective tumor killing (6, 7).

Magnetic nanoparticles (MNPs), particularly iron oxide nanoparticles (IONPs), are well suited to exploit this vulnerability. IONPs have long been studied for cancer-related applications because of their magnetic responsiveness, tunable surface chemistry, and biomedical versatility (8). They have also been associated with clinically relevant uses in cancer diagnosis and hyperthermia, which provides an important translational foundation for newer therapeutic designs (9, 10). Beyond their imaging and targeting value, MNPs can participate directly in ROS-mediated therapy by catalyzing redox reactions in the tumor milieu. Their surfaces can be engineered to improve stability, cellular uptake, and selective interaction with tumors, while their magnetic properties offer opportunities for external control and combination with other treatment modalities (11).

A key advantage of magnetic nanoparticle-based ROS therapy is its compatibility with the biochemical features of the tumor microenvironment. Solid tumors often exhibit acidity, elevated hydrogen peroxide levels, and abnormal redox homeostasis, all of which can be exploited to generate cytotoxic radicals locally (12). In particular, iron-containing nanomaterials can promote Fenton or Fenton-like reactions, converting endogenous oxidants into highly reactive hydroxyl radicals. This principle underlies chemodynamic therapy, a strategy in which catalytic nanoplatforms generate ROS *in situ* to induce oxidative injury preferentially inside tumors. Experimental work has shown that pH-responsive IONPs can be activated in acidic tumor settings, and iron-based nanoplatforms have been developed to enhance ROS production and reinforce oxidative cell death (13, 14).

ROS amplification by MNPs can engage multiple cell-death pathways. Excessive oxidative stress can damage membranes, DNA, and mitochondria, leading to cell-cycle arrest, apoptosis, lipid peroxidation, and ferroptosis. In iron-oxide-based systems, oxidative damage has been linked experimentally to DNA damage, lipid peroxidation, loss of mitochondrial potential, and reduced migratory capacity in cancer cells. These findings support the idea that MNPs do not act through a single mechanism, but rather through a network of interrelated oxidative and metabolic disruptions that can be therapeutically leveraged (15, 16).

Recent studies have also shown that MNPs can improve treatment response when combined with other therapeutic platforms. For example, iron-based nanostructures have been integrated with chemotherapy to intensify oxidative stress, with hyperthermia to accelerate redox injury, and with tumor-microenvironment-responsive designs to improve selectivity and efficacy (17). In some systems, MNPs have been shown to remodel the tumor microenvironment and delay resistant tumor emergence, while in others they have been used to strengthen chemodynamic therapy or induce synergistic ROS-mediated cytotoxicity (18). These advances indicate that magnetic nanoparticle-based ROS therapy is evolving from a single-modality concept into a broader theranostic framework. As illustrated in **Figure 1**, MNPs exploit the unique characteristics of the tumor microenvironment to induce ROS generation and activate multiple cell death pathways.

**Figure 1:**
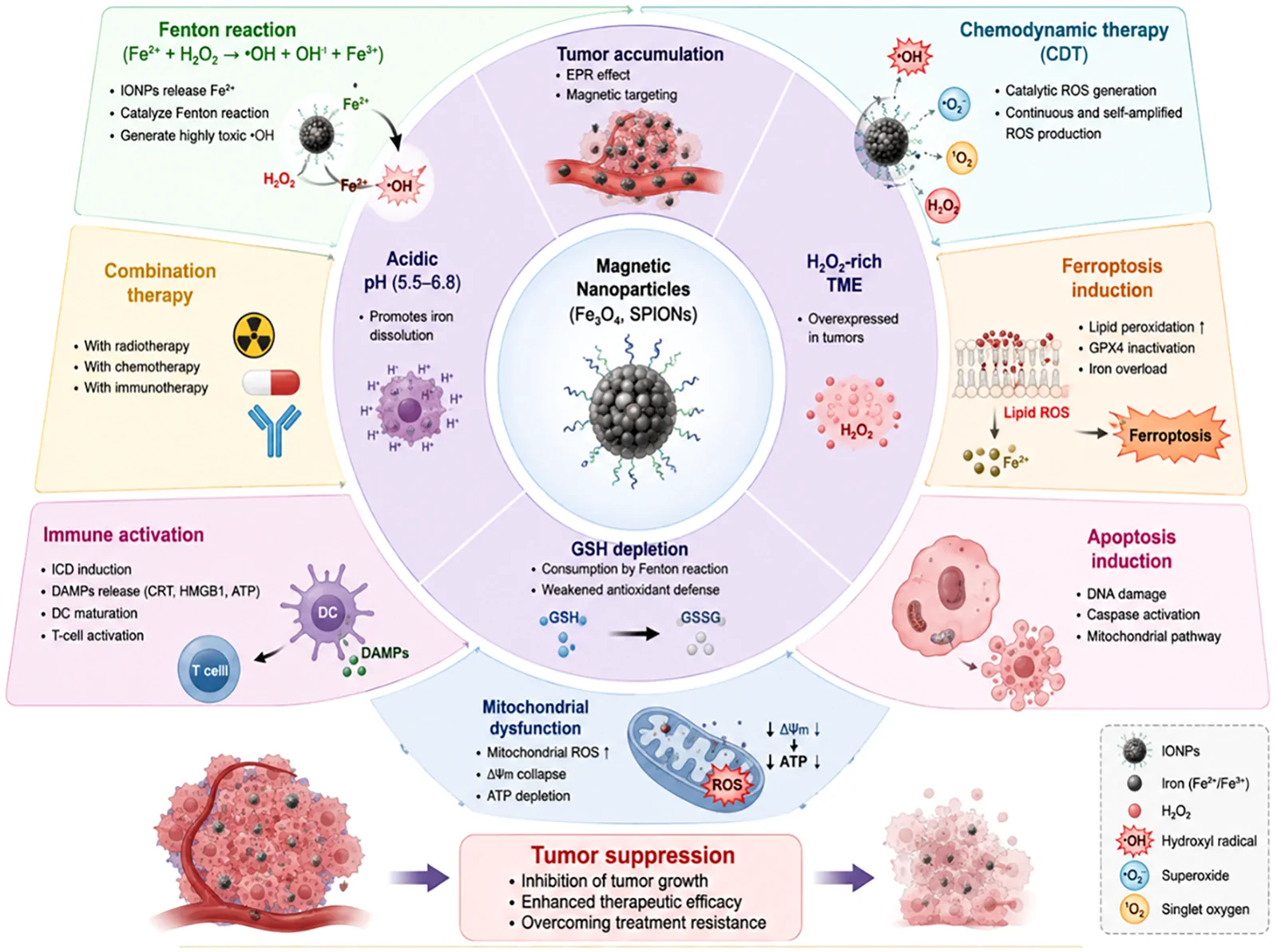
Schematic representation of ROS-mediated anticancer mechanisms induced by MNPs.

In contrast to reviews that mainly discuss chemodynamic therapy, ferroptosis, or magnetic nanomaterials as separate topics, this review adopts an integrated framework. It connects magnetic nanoparticle characteristics with tumor-responsive ROS generation and the resulting cell-death mechanisms. The review then links these mechanisms to rational engineering strategies, including surface functionalization, compositional optimization, targeted delivery, and stimuli-responsive design. It also examines how these strategies support combination treatments such as magnetic hyperthermia, photothermal therapy, photodynamic therapy, chemotherapy, and immunotherapy. This integrated sequence links nanoparticle design, ROS-generation mechanisms, engineering approaches, and therapeutic combinations within a single framework. It therefore provides a more connected view of how magnetic nanoplatform design can influence ROS amplification and downstream anticancer responses.

Despite these promising advances, important barriers remain before magnetic ROS-mediated nanotherapies can be translated into routine clinical use. Insufficient catalytic efficiency under some tumor conditions, heterogeneity in tumor oxygenation and pH, uncertain long-term biodistribution, and biosafety concerns continue to limit practical implementation. In addition, many systems still depend on favorable intratumoral conditions that are not uniform across patients or tumor types. Accordingly, this article summarizes the fundamental concepts, recent advances, and future directions of MNPs for ROS-mediated cancer therapy, with emphasis on how mechanistic understanding and rational nanoplatform engineering can be connected to multimodal therapeutic design and clinical translation.

## 2 Magnetic nanoparticles for ROS-mediated cancer therapy

MNPs have become an important class of nanomaterials in oncology because they combine magnetic responsiveness, tunable surface chemistry, and the ability to participate in redox-active processes relevant to tumor killing (19). In cancer therapy, their value goes beyond simple drug carriage. Iron-containing magnetic nanostructures can also act as catalytic platforms that exploit the biochemical vulnerabilities of tumors, especially the acidic and peroxide-rich tumor microenvironment. This makes them particularly attractive for ROS-mediated strategies such as chemodynamic therapy, where catalytic conversion of endogenous oxidants into highly toxic radicals is used to amplify local oxidative stress (20).

The therapeutic behavior of MNPs depends strongly on their composition, size, morphology, crystallinity, and surface modifications. These parameters influence not only colloidal stability and biodistribution, but also catalytic activity, cellular uptake, and the ability to generate ROS under tumor-relevant conditions (21). IONPs are especially notable because Fe^2+^/Fe^3+^ redox cycling can support Fenton or Fenton-like chemistry, thereby converting hydrogen peroxide into hydroxyl radicals and intensifying oxidative injury in cancer cells (22).

Another important reason for the growing interest in MNPs is their compatibility with the tumor microenvironment. Solid tumors often exhibit weak acidity, abnormal redox balance, elevated glutathione, and heterogeneous oxygen availability, all of which shape the performance of ROS-generating nanoplatforms. Rational nanodesign can therefore be used to respond to these local conditions and improve selectivity, while external magnetic guidance can further enhance tumor accumulation and reduce off-target exposure (23, 24).

Accordingly, this section first introduces the main types and characteristics of MNPs, then discusses how their physicochemical features govern ROS generation, and finally explains how tumor microenvironmental factors support or limit their anticancer activity. This framework provides the necessary background for understanding the mechanistic pathways and engineering strategies described in the following subsections.

### 2.1 Types and characteristics of magnetic nanoparticles

MNPs used in cancer therapy are not a uniform class of materials; their biological behavior depends on their core composition, magnetic state, and interfacial design. Among the most widely studied systems, magnetite (Fe_3_O_4_) and maghemite (γ-Fe_2_O_3_) dominate biomedical applications because they combine strong magnetic responsiveness with relatively favorable biocompatibility and broad opportunities for functionalization. These iron oxide cores are commonly engineered in the superparamagnetic size range, typically around 10–50 nm, which helps preserve magnetic responsiveness while minimizing unwanted aggregation after the external field is removed (25, 26).

In practice, the core is rarely used alone. Core–shell architectures are frequently adopted to improve colloidal stability, protect the magnetic core, and provide chemical handles for drug loading or ligand attachment (27). Surface layers such as polymers, silica, chitosan, polyethylene glycol, or other functional coatings can reduce nonspecific protein adsorption, improve dispersibility in physiological media, and moderate the interaction of the particle with healthy tissues. Coating choice is not a minor detail; it can reshape uptake, biodistribution, and the overall toxicity profile of the nanoparticle system (28, 29).

Particle size, surface charge, shell chemistry, and exposure dose also strongly influence how these materials behave in biological settings. Smaller particles may enter cells more readily, whereas larger or poorly stabilized systems can accumulate differently across organs and may produce distinct oxidative or inflammatory responses. Shape-controlled synthesis is particularly important because nanoscale geometry, surface structure, and macromolecule-mediated growth can strongly influence cellular interaction, colloidal behavior, and downstream biomedical activity (30). Because of this, two nanoparticles with similar iron content can still perform very differently in a tumor model if their coatings or hydrodynamic dimensions are changed. For ROS-mediated therapy, this point is especially important because the same structural features that improve catalytic access to the tumor microenvironment can also affect safety and clearance (31, 32). Ebadi et al. demonstrated that surface-engineered Fe_3_O_4_ nanoparticles possessed spherical morphology and relatively uniform particle dispersion according to TEM and FESEM observations, while XRD analysis confirmed the preservation of the crystalline magnetite structure following functionalization (**Figures 2a–c**) (33). Furthermore, Shi et al. reported that transferrin-functionalized PEG-PLGA MNPs exhibited a narrow hydrodynamic size distribution, supporting improved colloidal stability and enhanced tumor-targeting capability (**Figure 2d**) (34).

**Figure 2:**
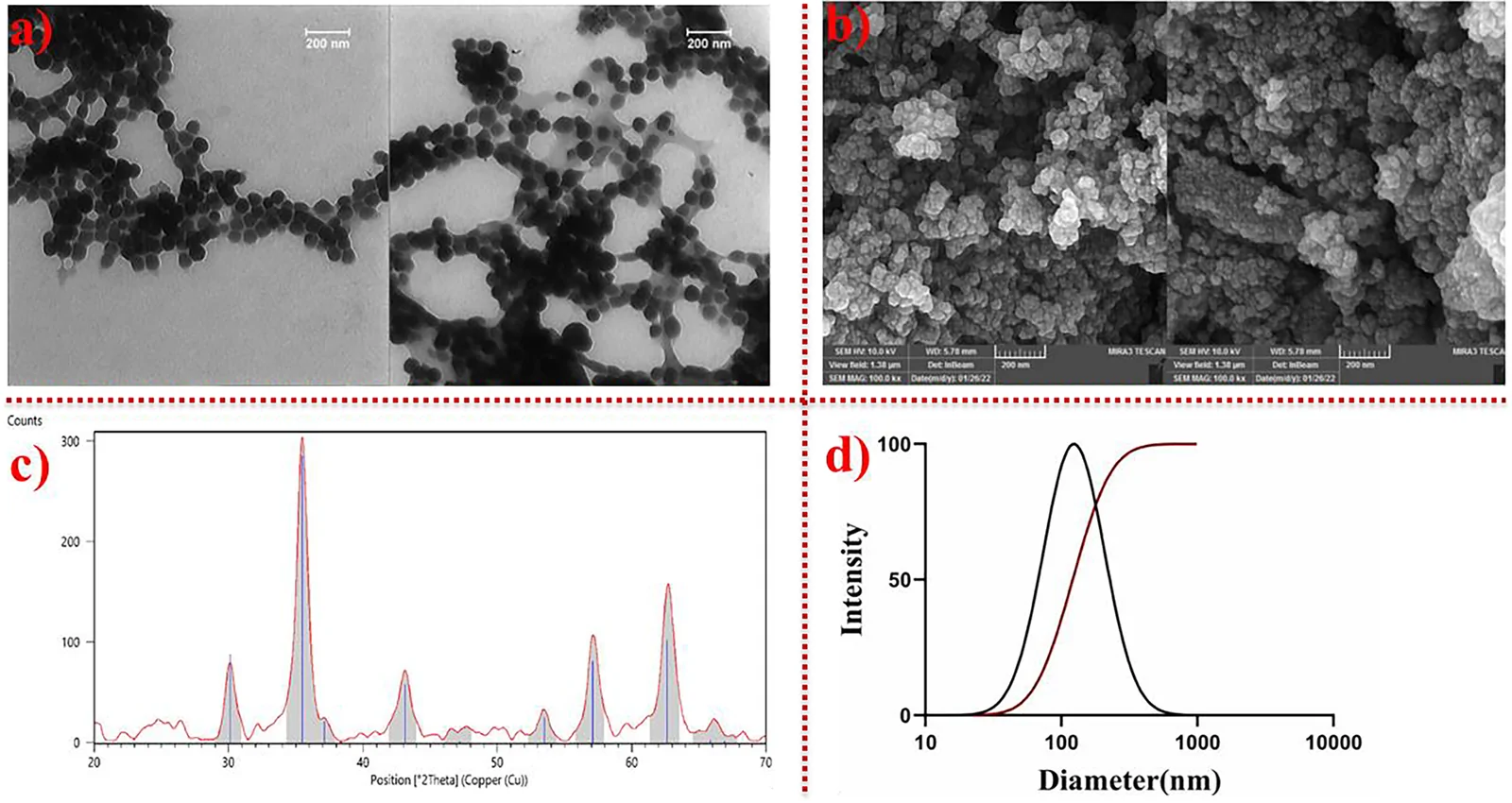
Representative physicochemical characterization of MNPs, including **(a)** TEM micrographs, **(b)** FESEM images, **(c)** XRD patterns. Reprinted with permission from ref (33); and **(d)** particle size distribution profiles of surface-engineered magnetic nanoplatforms. Reprinted with permission from ref (34). copyright *^©^ 2025 Published by Elsevier Ltd. License Number: 6300541167380*.

Taken together, the therapeutic potential of MNPs is determined by more than magnetism alone. Their clinical value emerges from a careful balance between composition, structure, surface engineering, and biological compatibility, which sets the stage for understanding how these properties influence ROS generation in the next subsection.

### 2.2 Tumor microenvironment and ROS generation

The tumor microenvironment (TME) is a highly dynamic and heterogeneous ecosystem composed of cancer cells, stromal cells, immune cells, extracellular matrix components, and a variety of biochemical factors that collectively influence tumor progression and therapeutic response (35). Unlike normal tissues, tumors frequently develop abnormal vascular networks that result in inadequate oxygen and nutrient delivery, creating regions characterized by hypoxia, acidity, and metabolic dysregulation. These unique conditions not only support malignant growth but also provide opportunities for the selective activation of ROS-generating nanotherapeutic systems (36). One of the most important biochemical features of the TME is the elevated concentration of hydrogen peroxide (H_2_O_2_), which originates from accelerated metabolism, mitochondrial dysfunction, and increased activity of oxidase enzymes in cancer cells (37). Although endogenous H_2_O_2_ levels are generally insufficient to cause extensive cytotoxicity on their own, they can serve as substrates for catalytic reactions mediated by iron-containing MNPs (23). Through Fenton or Fenton-like processes, relatively stable peroxide molecules can be transformed into highly reactive hydroxyl radicals (•OH), resulting in localized oxidative damage and enhanced therapeutic efficacy (38, 39).

Tumor acidity represents another critical factor affecting ROS production. Due to the predominance of aerobic glycolysis, commonly known as the Warburg effect, many solid tumors exhibit extracellular pH values lower than those found in healthy tissues. Acidic conditions facilitate iron ion release and accelerate catalytic redox reactions, thereby improving the efficiency of ROS generation. Consequently, magnetic nanoplatforms often display greater catalytic activity within tumor sites than in normal physiological environments, contributing to improved treatment selectivity (40, 41). Redox imbalance also plays a pivotal role in determining therapeutic outcomes. Cancer cells continuously produce elevated levels of ROS while simultaneously maintaining robust antioxidant defense systems, including glutathione (GSH), superoxide dismutase, and catalase (42). This fragile equilibrium allows tumor cells to survive under oxidative stress but leaves them vulnerable to additional ROS accumulation. Many advanced magnetic nanoplatforms are therefore designed not only to increase ROS generation but also to deplete intracellular antioxidants, further disrupting redox homeostasis and amplifying oxidative injury (23, 43).

Despite these advantages, the TME can also impose limitations on ROS-mediated therapy. Variations in pH, oxygen availability, antioxidant content, and peroxide concentration among different tumor types may significantly affect catalytic efficiency and therapeutic consistency. Therefore, understanding the complex interactions between MNPs and the tumor microenvironment is essential for the rational design of next-generation ROS-generating systems and for maximizing their anticancer potential.

Bai et al. demonstrated that nanozyme-based magnetic nanoplatforms could effectively exploit key biochemical features of the tumor microenvironment, including elevated H_2_O_2_ levels, glutathione overexpression, and hypoxia, to enhance ROS production and improve therapeutic outcomes (**Figure 3a**). In particular, intracellular H_2_O_2_ decomposition promoted oxygen generation and hypoxia alleviation, whereas glutathione depletion reduced ROS scavenging and facilitated oxidative stress amplification. These processes ultimately enhanced photodynamic therapy and stimulated CD4^+^ and CD8^+^ T-cell-mediated antitumor immune responses (44). In parallel, Zhao et al. further reported that Fe_3_O_4_-based magnetic nanoplatforms exhibited pH-responsive catalytic behavior within acidic tumor environments. Where Fe²^+^ ions catalyzed H_2_O_2_ decomposition via Fenton reactions to generate highly cytotoxic hydroxyl radicals (•OH) (**Figure 3b**). Simultaneously, catalase-like oxygen production alleviated hypoxia and improved photodynamic therapy efficiency. While photothermal heating further accelerated ROS generation and chemodynamic therapeutic efficacy under near-infrared irradiation (45).

**Figure 3:**
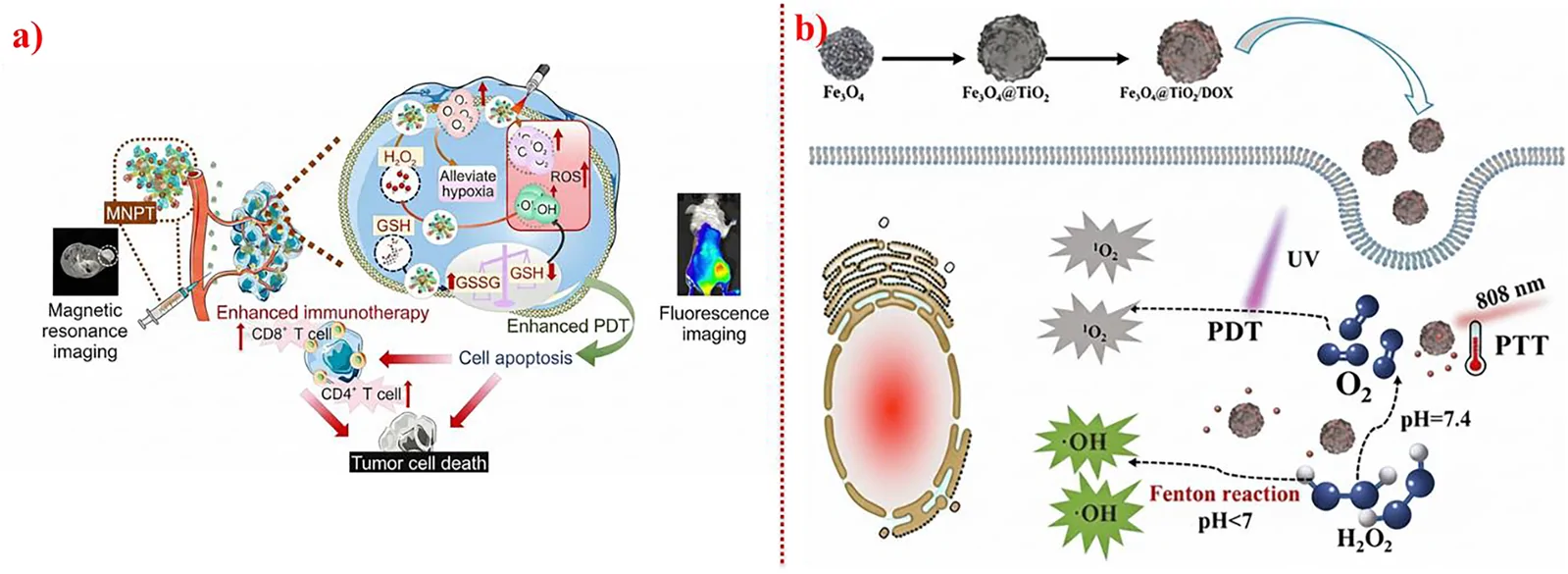
Tumor microenvironment-responsive mechanisms of ROS amplification in magnetic nanoplatform-mediated cancer therapy. **(a)** modulation of hypoxia, glutathione depletion, and enhanced photodynamic and immunotherapeutic responses. Reprinted with permission from ref (44). copyright ^©^ 2023 The Authors. Published by Elsevier B.V. on behalf of Xi’an Jiaotong University; and **(b)** pH-responsive Fenton reactions, oxygen generation, and synergistic CDT/PDT/PTT effects in Fe_3_O_4_-based magnetic nanoplatforms. Reprinted with permission from ref (45).

### 2.3 Factors influencing ROS production

The efficiency of ROS-mediated cancer therapy is closely linked to the catalytic performance of MNPs, which is governed by multiple physicochemical and biological parameters. Even nanoparticles composed of the same magnetic material may exhibit substantially different ROS-generating capabilities due to variations in structural and surface characteristics. Therefore, understanding the factors that regulate ROS production is essential for optimizing therapeutic efficacy and minimizing undesirable side effects (46, 47). Particle size is one of the most influential parameters affecting catalytic activity. Reducing particle dimensions generally increases the surface-to-volume ratio, exposing a larger number of active sites for redox reactions (48). Smaller nanoparticles may also facilitate intracellular internalization, thereby increasing contact with endogenous substrates such as hydrogen peroxide. However, excessive size reduction can compromise magnetic properties, alter biodistribution patterns, and accelerate systemic clearance, highlighting the need for careful size optimization (49).

Morphology and crystal structure also contribute significantly to catalytic behavior. Nanoparticles with distinct geometries, including spheres, cubes, rods, and flower-like structures, often exhibit different surface atom arrangements and exposed crystal facets. These structural variations influence electron transfer processes and iron ion accessibility, both of which directly affect ROS generation efficiency. Furthermore, crystal defects and lattice imperfections may create additional catalytic sites that enhance redox activity (50, 51). Surface chemistry represents another critical determinant of ROS production. Functional coatings not only improve nanoparticle stability but also regulate interactions with biological environments (52). Surface modifications can influence cellular uptake, protein corona formation, intracellular trafficking, and catalytic accessibility. In addition, certain coatings are specifically designed to respond to tumor-associated stimuli such as acidic pH, elevated glutathione concentrations, or enzyme overexpression, allowing ROS generation to occur preferentially within malignant tissues (53).

The chemical composition of MNPs further affects their catalytic potential. Incorporating transition metals such as manganese, cobalt, copper, or nickel into iron-based structures can alter electronic properties and accelerate redox cycling (54). Such compositional engineering strategies are increasingly employed to enhance Fenton-like reactions, improve catalytic efficiency, and overcome limitations associated with insufficient endogenous ROS generation (55, 56). Beyond nanoparticle-related factors, biological conditions within the tumor environment also strongly influence ROS production. Local hydrogen peroxide availability, pH values, oxygen levels, antioxidant concentrations, and metabolic activity collectively determine the extent to which catalytic reactions can proceed (57). Consequently, the therapeutic performance of magnetic nanoplatforms depends not only on material design but also on their ability to adapt to the heterogeneous and dynamic nature of tumor tissues.

The therapeutic efficiency of ROS-mediated magnetic nanoplatforms strongly depends on multiple interconnected factors, including nanozyme activity, H_2_O_2_ utilization efficiency, magnetic responsiveness, and tumor microenvironmental conditions. Recent studies have reported substantial variations in catalytic performance and anticancer efficacy according to these parameters, highlighting the importance of rational nanoplatform design for maximizing ROS production and therapeutic outcomes (**Table 1**).

**Table 1:** Quantitative parameters influencing ROS generation in magnetic nanoplatforms.

Magnetic nanoplatform	Quantitative parameter	Value	ROS-related effect	Ref.
A-MPDA@Fe_3_O_4_@PVP	Fe_3_O_4_ size	Ultra-small	Enhanced CAT/SOD-mimicking ROS scavenging activity	(58)
Fe_3_O_4_ nanoparticles	NP concentration	50 mg L^-^¹	Increased oxidative stress signaling	(59)
		100 mg L^-^¹	Enhanced CAT, SOD, POX, and PAL activity	
		200 mg L^-^¹	Maximum ROS-associated metabolic response	
CUR/CAPE-loaded SPION nanoclusters	IC_50_ in MCF-7 cells	0.43 μg mL^-^¹	CAP-mediated ROS amplification	(60)
	IC_50_ in HME-1 cells	37 μg mL^-^¹	Reduced toxicity toward normal cells	
Fe_3_O_4_@NC nanozyme	Increase in POD activity	42.7%	Improved Fenton catalytic activity	(61)
	Increase in Vmax	~6-fold	Enhanced H_2_O_2_ utilization efficiency	
Ferrocene/P-Fe_2_O_3_ microspheres	Trigger type	ROS + magnetic field	Dual-responsive drug release	(62)

Overall, ROS generation is governed by a complex interplay between nanoparticle properties and tumor-specific biological conditions. Rational control of these variables provides a foundation for designing highly efficient magnetic nanoplatforms capable of achieving precise and amplified oxidative damage in cancer cells.

## 3 Mechanisms of ROS-mediated cancer therapy

ROS play a central role in oxidative cancer therapy by disrupting cellular redox homeostasis and inducing irreversible molecular damage. Compared with normal cells, cancer cells often maintain elevated basal ROS levels and therefore exhibit increased susceptibility to further oxidative stress. MNPs provide a versatile platform for amplifying ROS generation within tumors through catalytic redox reactions and tumor microenvironment-responsive mechanisms. Depending on nanoparticle composition and local biological conditions, ROS overproduction can activate multiple cell death pathways, including chemodynamic therapy, ferroptosis, apoptosis, and immune-related responses. Understanding these mechanisms is essential for optimizing therapeutic efficacy and guiding the development of advanced magnetic nanoplatforms. The following sections discuss the major ROS-mediated anticancer mechanisms associated with MNPs.

### 3.1 Fenton and Fenton-like reactions

Among the various ROS-generating mechanisms employed in cancer nanotherapy, Fenton and Fenton-like reactions represent some of the most extensively investigated catalytic processes. These reactions are based on the conversion of relatively weak oxidants into highly reactive radical species capable of causing severe intracellular damage. Their importance stems from the ability to exploit endogenous biochemical substrates within tumors, thereby reducing dependence on external energy sources and enabling localized oxidative injury (63, 64).

The classical Fenton reaction involves the interaction between ferrous ions (Fe²^+^) and hydrogen peroxide (H_2_O_2_), leading to the formation of hydroxyl radicals (•OH), which are among the most reactive oxygen-derived species found in biological systems. The reaction is commonly written as Equation 1:

(1) $$Fe^{2+}+H_{2}O_{2}\to Fe^{3+}+•OH+OH^{-}$$

Due to their extremely short lifetime and high oxidation potential, hydroxyl radicals rapidly react with nearby biomolecules, triggering lipid peroxidation, protein oxidation, DNA strand breaks, and organelle dysfunction. As a consequence, even modest increases in hydroxyl radical production can substantially compromise cancer cell viability (65, 66).

Iron-containing MNPs serve as effective catalytic reservoirs for these reactions. Following cellular internalization or degradation within acidic intracellular compartments, iron ions may be released from the nanoparticle structure and participate in continuous redox cycling. This process replenishes catalytically active iron species and sustains ROS generation over extended periods. In addition, the large surface area of nanostructured materials provides abundant catalytic interfaces, facilitating interactions between iron centers and endogenous peroxide molecules (20, 67). A representative Fenton-like regeneration step is often expressed as Equation 2:

(2) $$Fe^{3+}+H_{2}O_{2}\to Fe^{2+}+HO_{2}•+H^{+}$$

Although the traditional Fenton reaction relies on Fe²^+^, numerous Fenton-like systems have been developed to overcome limitations associated with catalytic efficiency. In these systems, transition metals such as manganese, copper, cobalt, or mixed-metal architectures participate in peroxide activation through alternative redox pathways. Such modifications can broaden the operational pH range, accelerate electron transfer, and improve ROS yield under physiological conditions. Consequently, Fenton-like catalysts have become increasingly attractive for enhancing oxidative therapy in tumors where conventional iron-mediated reactions may be less effective (68, 69).

A major limitation of conventional iron-mediated Fenton chemistry is the inefficient regeneration of catalytically active Fe²^+^. Although Fe²^+^ readily activates H_2_O_2_ to generate hydroxyl radicals, the subsequent Fe³^+^/Fe²^+^ recycling is considerably slower and can therefore restrict sustained radical production. Strategies that accelerate electron transfer or facilitate Fe²^+^ regeneration can partially overcome this kinetic bottleneck (70). In addition, Fenton chemistry in tumors is limited by substrate availability. The intracellular H_2_O_2_ concentration in cancer cells is generally reported within the micromolar range, approximately 10–100 μM, which can be insufficient to maintain high-flux ROS production during prolonged CDT. Thus, improving catalytic turnover alone may not fully compensate for limited peroxide availability (71).

Transition-metal incorporation provides an alternative strategy for improving catalytic kinetics because Cu-, Mn-, and Co-based redox couples can activate H_2_O_2_ through pathways that differ from the conventional Fe²^+^/Fe³^+^ cycle. However, their catalytic behavior depends strongly on oxidation state, coordination environment, and nanostructure. Heterojunction engineering can further improve catalytic turnover by promoting directional interfacial electron transfer. For example, a Fe@Fe_3_O_4_-Cu_2_O heterostructure with a built-in electric field maintained continuous Fenton-like •OH production for more than 18 h through sustained reduction of surface Cu²^+^ to Cu^+^ (72). Local heating represents another complementary strategy. Combined photothermal and magnetic hyperthermia can accelerate temperature-dependent Fenton reactions and enhance ROS production within the tumor microenvironment, thereby improving CDT efficiency without relying solely on increasing catalyst concentration (73). As summarized in **Table 2**, conventional Fe-based Fenton chemistry is constrained by slow metal-ion recycling and limited H_2_O_2_ availability, whereas alternative metal centers, interfacial electron-transfer engineering, and localized thermal activation provide complementary routes for improving catalytic kinetics. Also, solution-phase rate constants should be regarded as mechanistic benchmarks rather than universal values for nanoparticle surfaces, because apparent catalytic rates are strongly influenced by coordination chemistry, surface structure, pH, and the local reaction environment.

**Table 2:** Representative kinetic characteristics and strategies for overcoming catalytic limitations in Fenton and Fenton-like systems relevant to chemodynamic cancer therapy.

Catalytic system	Representative kinetic characteristic	Major implication for CDT	Kinetic enhancement strategy	Ref.
Fe²^+^/Fe³^+^ versus Cu^+^/Cu²^+^ redox chemistry	Fe²^+^ + H_2_O_2_: k ≈ 63–76 M^-^¹ s^-^¹; Fe³^+^/H_2_O_2_-mediated Fe²^+^ regeneration: k ≈ 0.001–0.02 M^-^¹ s^-^¹. Cu^+^ + H_2_O_2_ is markedly faster, with k ≈ 1.0 × 10^4^ M^-^¹ s^-^¹, while Cu²^+^/H_2_O_2_ reduction has been reported at ~4.6 × 10² M^-^¹ s^-^¹ under representative solution conditions.	Slow Fe³^+^ reduction can limit sustained Fe²^+^ availability, whereas Cu cycling can provide faster peroxide activation and low-valence metal regeneration.	Incorporation of Cu into Fe-containing or hybrid catalytic systems to accelerate redox cycling.	(78)
Mn-based Fenton-like systems	Mn²^+^/Mn³^+^/Mn^4+^ redox behavior is strongly dependent on oxide structure, local coordination, and the tumor environment rather than a single transferable rate constant.	Mn-based catalysts can provide additional redox pathways and support Fenton-like ROS production, particularly after tumor-responsive Mn ion release.	Mn doping, MnO_2_ decomposition, and mixed-metal nanostructures.	(79)
Co-based Fenton-like systems	The activity of Co²^+^ toward H_2_O_2_ is highly coordination-dependent. Ligand and second-sphere interactions can alter H_2_O_2_ activation and determine the dominant ROS pathway.	Catalytic performance cannot be predicted from Co content alone and requires control of the local coordination environment.	Coordination engineering and controlled Co active-site design.	(80)
Heterojunction-assisted catalysis	Built-in electric fields promote directional carrier separation and interfacial electron transfer, increasing the availability of catalytically active charge carriers.	Improved electron transfer can reduce redox-cycling limitations and strengthen Fenton-like ROS generation.	Band and interface engineering of heterojunction nanoplatforms.	(81)
MHT-assisted Fenton catalysis	Localized temperature elevation accelerates Fenton and Fenton-like reaction kinetics and increases ROS generation.	Thermal activation can increase catalytic turnover where room-temperature reaction kinetics are insufficient.	Integration of magnetic hyperthermia with CDT.	(82)

The therapeutic outcome of these catalytic processes is strongly influenced by the local biochemical environment. Factors such as peroxide availability, intracellular iron concentration, redox status, and pH conditions determine the extent of radical generation and subsequent biological damage. Tumor tissues often provide a more favorable catalytic setting than healthy tissues, enabling selective oxidative stress amplification and reducing nonspecific toxicity (74, 75). Wei et al. demonstrated that magnetic Fe-based nanocomplexes enhance ROS-mediated tumor destruction through magnetic targeting, photothermal activation, Fe³^+^/Fe²^+^ cycling, and ferroptosis amplification (**Figure 4a**) (76). In parallel, Fu et al. designed GOx-assisted Fenton nanoplatforms capable of generating endogenous H_2_O_2_ and promoting synergistic apoptosis and ferroptosis via cascade catalytic reactions (**Figure 4b**) (77).

**Figure 4:**
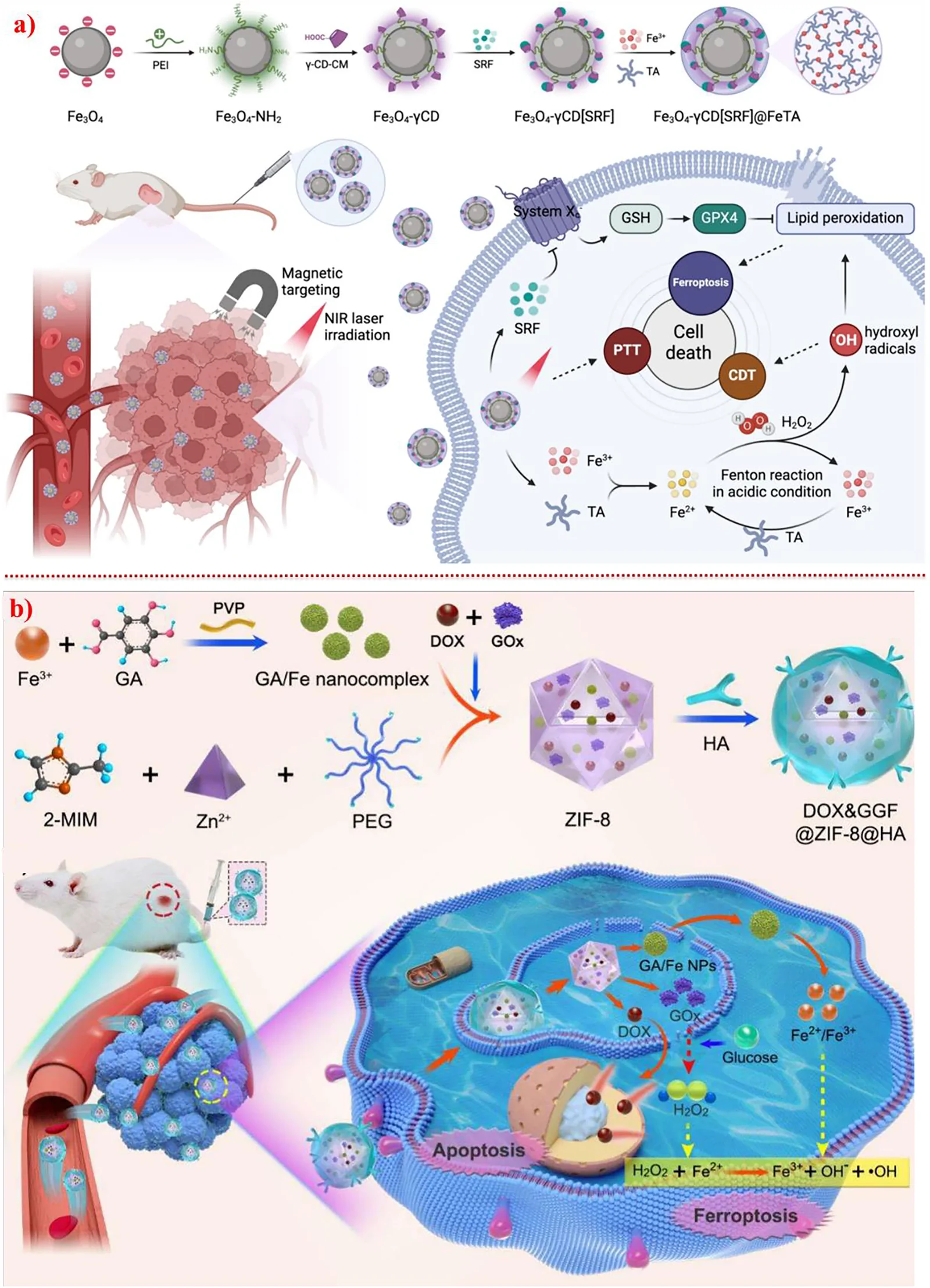
Representative Fenton-based magnetic nanoplatforms for ROS-mediated cancer therapy and ferroptosis enhancement. **(a)** Magnetic Fe-based nanocomplexes for combined chemodynamic and photothermal therapy through Fe³^+^/Fe²^+^ cycling and ferroptosis amplification. Reprinted with permission from ref (76). copyright *^©^ 2024 Elsevier B.V. All rights are reserved, including those for text and data mining, AI training, and similar technologies. License Number: 6300550609230.* **(b)** GOx-assisted cascade nanoplatforms for endogenous H_2_O_2_ generation, Fenton reaction enhancement, and synergistic apoptosis–ferroptosis therapy. Reprinted with permission from ref (77). copyright *^©^ 2021 Elsevier Ltd. All rights reserved. License Number: 6300550943198*.

Recent advances have focused on engineering magnetic nanoplatforms capable of improving catalytic performance through structural optimization, elemental doping, and stimulus-responsive designs. These approaches aim to maximize radical production while minimizing systemic side effects, ultimately enhancing the precision and efficacy of ROS-mediated cancer therapy. As a result, Fenton and Fenton-like reactions remain fundamental mechanistic pillars underpinning the development of next-generation magnetic nanotherapeutics.

### 3.2 Chemodynamic therapy

Chemodynamic therapy (CDT) is a catalytic therapeutic strategy that exploits the unique biochemical characteristics of tumors to generate cytotoxic reactive oxygen species *in situ*. Unlike conventional treatment modalities that rely on externally applied energy sources, CDT utilizes endogenous chemical substrates already present within the tumor microenvironment to induce oxidative damage. This approach has attracted considerable attention because it enables localized therapeutic action while minimizing dependence on external stimuli (55, 83). The fundamental principle of CDT is based on catalytic reactions that convert relatively stable intracellular molecules into highly reactive radical species. In most cases, transition-metal-containing nanomaterials activate endogenous hydrogen peroxide and trigger oxidative processes capable of damaging essential cellular structures. As a result, cancer cells experience severe oxidative stress, leading to disruption of biological functions and loss of viability. Because these reactions occur directly within tumor tissues, CDT offers a potentially selective strategy for cancer treatment (84, 85).

MNPs are among the most extensively investigated catalysts for CDT due to their iron-rich composition and favorable catalytic properties. Following accumulation within tumors, these nanomaterials can serve as continuous sources of catalytic metal ions that promote ROS generation under biologically relevant conditions. Their nanoscale dimensions also facilitate cellular internalization and increase contact between catalytic sites and intracellular substrates, thereby improving reaction efficiency (86, 87). The tumor microenvironment plays a central role in determining CDT performance. Elevated peroxide concentrations, mild acidity, altered metabolic activity, and disturbed redox homeostasis collectively create conditions that favor catalytic ROS production. These biochemical features distinguish malignant tissues from healthy organs and provide a basis for selective oxidative injury. Consequently, CDT is often considered a tumor-microenvironment-responsive therapeutic strategy rather than a purely material-dependent treatment (88).

Endogenous hydrogen peroxide levels may be insufficient to sustain robust radical production in some tumors. Intratumoral H_2_O_2_ concentrations are commonly reported in the approximate range of 50–100 μM, which may not provide sufficient substrate flux for sustained high-efficiency Fenton chemistry (89). High concentrations of intracellular antioxidants can neutralize ROS before substantial damage occurs. In addition, variations in pH and metabolic characteristics among different tumor types may lead to inconsistent catalytic activity. These challenges have motivated the development of advanced nanoplatforms designed to enhance catalytic efficiency and overcome microenvironmental constraints (90, 91). Recent research has focused on improving CDT through rational material engineering. Approaches such as increasing catalytic active sites, incorporating secondary catalytic metals, accelerating electron-transfer processes, and designing tumor-responsive nanostructures have demonstrated enhanced ROS production and improved therapeutic outcomes. Furthermore, multifunctional magnetic nanoplatforms capable of simultaneously modulating tumor biochemistry and promoting catalytic reactions have shown considerable potential for increasing treatment efficacy (86, 92).

Overall, CDT represents one of the most important ROS-mediated therapeutic mechanisms associated with MNPs. By utilizing endogenous tumor chemistry as a source of catalytic activation, this strategy provides a foundation for selective oxidative destruction of cancer cells and continues to drive the development of next-generation nanocatalytic therapeutics.

### 3.3 Ferroptosis-induced cancer cell death

Ferroptosis is a regulated form of cell death characterized by excessive lipid peroxidation and the loss of membrane integrity resulting from iron-dependent oxidative reactions. Unlike apoptosis, necrosis, or autophagy, ferroptosis is primarily driven by the uncontrolled accumulation of lipid hydroperoxides within cellular membranes. Because iron plays a central role in both ROS generation and lipid oxidation, ferroptosis has become one of the most important mechanisms linking MNPs to cancer therapy (93–95). The initiation of ferroptosis is closely associated with disturbances in cellular antioxidant defense systems. Under normal physiological conditions, enzymes such as glutathione peroxidase 4 (GPX4) protect membrane lipids from oxidative damage by reducing lipid hydroperoxides to less harmful products. This GPX4-dependent detoxification reaction consumes reduced GSH and converts it to oxidized glutathione (GSSG), thereby limiting the accumulation of phospholipid hydroperoxides as Equation 3:

(3) $$\text{PL}-\text{OOH}+2\;\text{GSH}\overset{GPX4}{\to }\text{PL}-\text{OH}+\text{GSSG}+{\text{H}}_{2}\text{O}$$

When this protective machinery becomes impaired, oxidized lipids progressively accumulate and ultimately reach toxic levels. As a consequence, membrane structure and function deteriorate, leading to irreversible cellular damage and death (96, 97).

Iron availability is a key factor governing ferroptotic sensitivity. Elevated intracellular iron can accelerate oxidative reactions through redox cycling and promote the formation of reactive radical species capable of initiating lipid peroxidation. Because MNPs often contain iron-rich cores, they can contribute directly to intracellular iron pools following degradation or dissolution within cellular compartments. This additional iron supply can enhance oxidative damage and facilitate ferroptotic processes in tumor cells (98, 99). Lipid metabolism also plays a critical role in ferroptosis. Polyunsaturated fatty acids present within membrane phospholipids are particularly susceptible to oxidation, making them major targets during ferroptotic progression (100). Once oxidative chain reactions begin, lipid radicals propagate rapidly throughout the membrane, amplifying cellular damage. Consequently, cancer cells with active lipid metabolism or elevated polyunsaturated lipid content may exhibit increased vulnerability to ferroptosis-inducing therapies (101). One of the major advantages of ferroptosis-based treatment is its potential to overcome resistance associated with conventional anticancer therapies. Many tumors that evade apoptosis remain susceptible to lipid peroxidation-mediated death pathways. This distinction has generated significant interest in combining ferroptosis induction with ROS-generating nanomaterials to improve treatment efficacy against aggressive or drug-resistant malignancies (100, 102).

Huang et al. demonstrated that CM-Fe-siR nanohybrids significantly enhanced ferroptotic cell death by increasing cytotoxicity and reducing cellular viability compared with control groups (**Figure 5a(i and ii)**). Furthermore, the substantial decrease in red/green fluorescence ratios indicated pronounced lipid peroxidation, while cell cycle analysis revealed marked G2 phase arrest following treatment with CM-Fe-siR nanoparticles (**Figure 5a(iii and iv)**). Mechanistically, iron ions released from the nanoplatform promoted Fenton reactions and intracellular ROS accumulation, whereas RNA interference-mediated SLC7A11 silencing inhibited cystine uptake and depleted intracellular glutathione levels (**Figure 5b**). The subsequent inactivation of GPX4 impaired lipid peroxide detoxification and ultimately accelerated ferroptotic cell death through excessive lipid peroxidation (103). In parallel, Xie et al. reported that PEG-Fe-MOF nanoplatforms induced concentration-dependent ferroptosis and significantly enhanced therapeutic efficacy under NIR irradiation. Flow cytometric analysis confirmed increased cell death with increasing nanoparticle concentrations, whereas NIR-assisted treatment further reduced tumor cell viability owing to synergistic ROS amplification and photodynamic enhancement (**Figures 5c, d**) (104).

**Figure 5:**
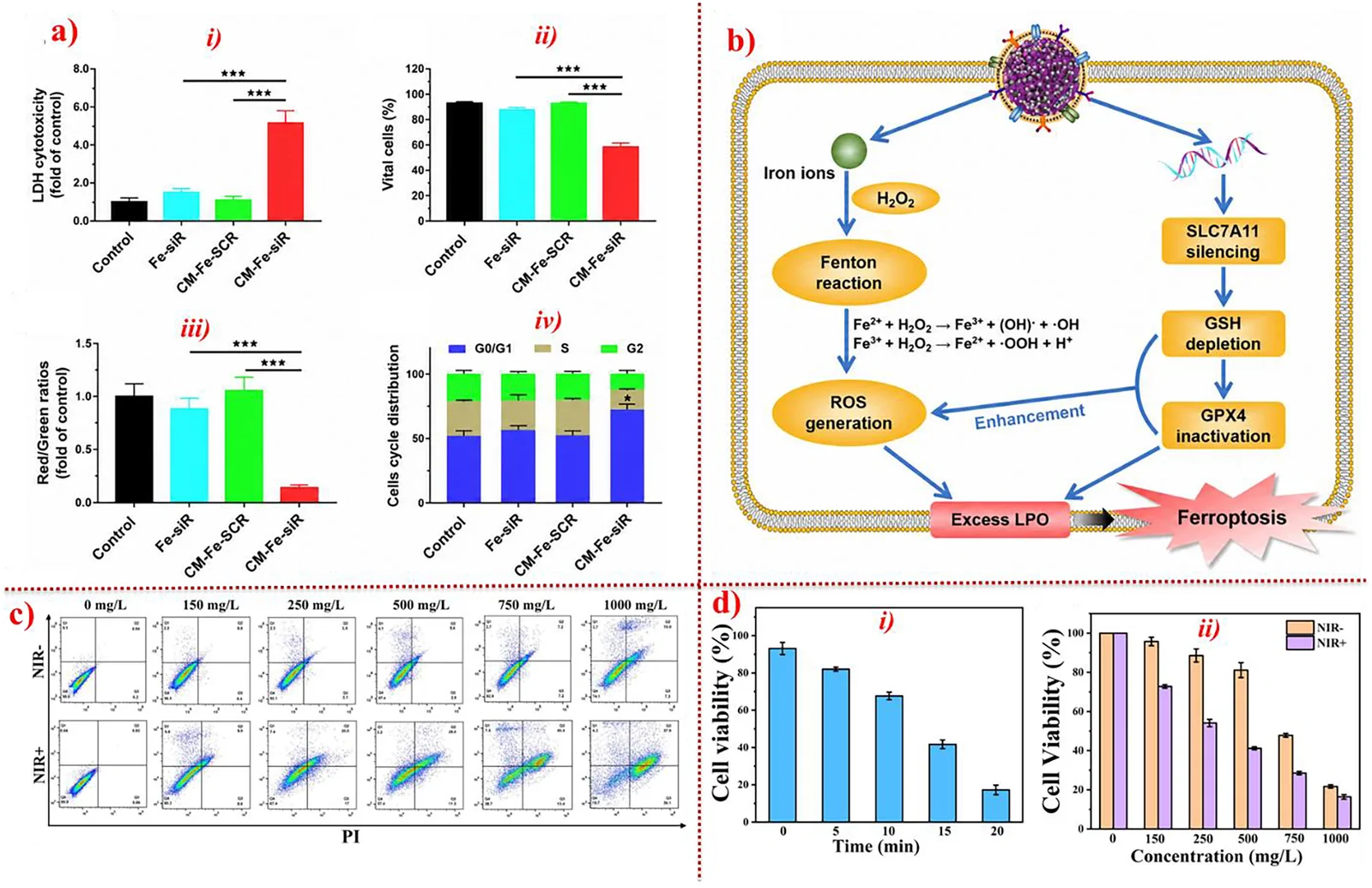
Representative experimental evidence of iron-mediated ferroptosis induction through ROS amplification and antioxidant disruption. **(a)** Cytotoxicity, cell viability, lipid peroxidation, and cell cycle alterations induced by CM-Fe-siR nanohybrids. **(b)** Mechanistic pathway of ferroptosis involving Fenton reaction-mediated ROS production, SLC7A11 silencing, glutathione depletion, GPX4 inactivation, and lipid peroxide accumulation. Reprinted with permission from ref (103). copyright ^©^ 2022 Acta Materialia Inc. Published by Elsevier Ltd. All rights reserved. License Number: 6300560030352. **(c)** Flow cytometric analysis of ferroptotic cell death under different nanoparticle concentrations and NIR irradiation conditions. **(d)** Time- and concentration-dependent therapeutic efficacy of PEG-Fe-MOF nanoplatforms with and without NIR activation. Reprinted with permission from ref (104). copyright ^©^ 2025 Elsevier Inc. All rights are reserved, including those for text and data mining, AI training, and similar technologies. License Number: 6300560379855.

Recent advances have focused on designing magnetic nanoplatforms capable of simultaneously enhancing iron availability, promoting ROS formation, and disrupting antioxidant defense mechanisms. Such multifunctional systems can amplify lipid peroxidation and strengthen ferroptotic responses within tumors. As understanding of ferroptosis continues to expand, this mechanism is increasingly recognized as a promising therapeutic pathway for improving the effectiveness of ROS-mediated cancer treatment.

### 3.4 Apoptosis and mitochondrial damage

Mitochondria are essential organelles responsible for energy production, metabolic regulation, and cellular survival. Beyond their role in bioenergetics, they function as critical regulators of programmed cell death and are highly sensitive to oxidative disturbances (105). Consequently, excessive ROS accumulation can profoundly affect mitochondrial integrity and initiate signaling pathways that culminate in apoptosis. In ROS-mediated cancer therapy, mitochondrial dysfunction therefore represents a major route through which MNPs exert their therapeutic effects (106). One of the earliest consequences of oxidative stress is the disruption of mitochondrial membrane integrity. Elevated ROS levels can damage mitochondrial lipids, proteins, and mitochondrial DNA, impairing normal respiratory chain activity (107). As electron transport becomes compromised, additional ROS may be generated, creating a self-amplifying cycle of oxidative injury. This progressive deterioration ultimately weakens mitochondrial function and destabilizes intracellular homeostasis (108, 109).

Mitochondrial membrane depolarization is another hallmark of oxidative damage. Under physiological conditions, the mitochondrial membrane potential is carefully maintained to support ATP synthesis and metabolic activity (110). However, excessive oxidative stress can trigger permeability alterations that dissipate this electrochemical gradient. Loss of membrane potential is frequently accompanied by increased membrane permeability and the release of pro-death signaling molecules into the cytoplasm (111, 112). Among the most important mediators released from damaged mitochondria is cytochrome *c*. Once translocated into the cytosol, cytochrome *c* participates in the formation of multiprotein complexes that activate downstream caspases, leading to the execution of apoptosis (113, 114). This pathway is tightly regulated by members of the Bcl-2 protein family, which control mitochondrial membrane permeability and determine cellular susceptibility to apoptotic stimuli. As a result, oxidative mitochondrial injury often serves as a critical trigger for programmed cancer cell elimination (115). MNPs can promote these events through sustained ROS generation and intracellular redox disruption. Following cellular uptake, catalytic oxidative reactions may damage mitochondrial structures directly or indirectly through secondary radical species (25, 106). Several studies have demonstrated that iron-containing nanomaterials can induce mitochondrial swelling, membrane depolarization, ATP depletion, and activation of apoptotic signaling cascades, ultimately reducing tumor cell viability (116).

Unlike ferroptosis, which is primarily associated with lipid peroxidation, apoptosis involves a coordinated sequence of molecular events leading to controlled cellular dismantling. Nevertheless, these pathways are not entirely independent. Oxidative stress generated by MNPs can simultaneously influence mitochondrial function, apoptotic signaling, and other forms of regulated cell death, creating a multifaceted therapeutic response. Therefore, mitochondrial dysfunction and apoptosis remain key contributors to the overall anticancer activity of ROS-generating magnetic nanoplatforms.

### 3.5 ROS-mediated immune activation

Beyond their direct cytotoxic effects, reactive oxygen species can influence the interaction between tumors and the immune system. Oxidative stimuli generated during cancer treatment may alter cellular signaling, promote the release of immunologically active molecules, and reshape the immune landscape within tumor tissues. As a result, ROS are increasingly recognized not only as mediators of cancer cell destruction but also as regulators of antitumor immunity (117, 118). A particularly important consequence of oxidative injury is the induction of immunogenic cell death (ICD). Unlike non-immunogenic forms of cell elimination, ICD is accompanied by the exposure or release of danger-associated molecular patterns (DAMPs), including calreticulin, ATP, and high-mobility group box 1 (HMGB1). These signals function as molecular alarms that attract and activate antigen-presenting cells, thereby facilitating the recognition of tumor-associated antigens by the immune system (119, 120).

Dendritic cells play a central role in this process. Following exposure to DAMPs and tumor-derived antigens, dendritic cells undergo maturation and migrate to lymphoid tissues, where they initiate adaptive immune responses through T-cell priming (121). Consequently, localized oxidative damage may extend beyond the directly treated region and contribute to systemic immune surveillance against residual malignant cells. ROS-associated immune stimulation can also affect the composition and activity of immune populations within the tumor microenvironment. Enhanced antigen presentation, increased cytotoxic T-lymphocyte infiltration, and modulation of macrophage polarization have all been reported following oxidative stress-based therapies. These changes may help shift the tumor milieu from an immunosuppressive state toward a more immunologically active environment capable of supporting antitumor responses (122, 123).

MNPs offer unique opportunities to enhance these immune-related effects. Their ability to generate oxidative stress in a controlled manner, combined with their capacity for surface functionalization, enables the development of multifunctional systems that integrate catalytic therapy with immune modulation. In some designs, ROS generation is intentionally combined with immune-stimulatory agents to amplify antigen release and strengthen immune recognition of tumor cells (20, 124). Nevertheless, the relationship between ROS and immunity remains complex. While moderate oxidative stimulation can support immune activation, excessive or prolonged oxidative stress may impair immune-cell viability and function. Therefore, achieving an appropriate balance between cytotoxicity and immune stimulation represents an important consideration in the design of ROS-mediated nanotherapeutic platforms.

Overall, ROS-mediated immune activation provides an additional therapeutic dimension beyond direct tumor destruction. By promoting immunogenic signaling, facilitating antigen presentation, and supporting adaptive immune responses, oxidative stress-generating MNPs may contribute to both local tumor control and broader systemic anticancer immunity.

### 3.6 Cross-talk and mechanistic prioritization among ROS-mediated pathways

Although CDT, ferroptosis, apoptosis, and immune activation are commonly described as distinct processes, their functions in magnetic nanoparticle-mediated therapy are closely interconnected. CDT primarily represents an upstream catalytic process rather than an independent form of regulated cell death. Its efficiency is controlled by H_2_O_2_ availability, local acidity, catalytic metal cycling, and antioxidant buffering. Increasing H_2_O_2_ supply, lowering local pH, or accelerating Fe³^+^/Fe²^+^ cycling can strengthen Fenton chemistry and increase the oxidative pressure imposed on tumor cells. However, these physicochemical conditions alone do not determine the final cell-death phenotype (71).

The transition from ROS accumulation to ferroptosis is mainly determined by whether oxidative stress can overcome lipid-peroxide defense systems. Elevated labile iron promotes radical formation and lipid oxidation, while suppression of the SLC7A11/GSH/GPX4 axis reduces the capacity of cells to detoxify phospholipid hydroperoxides. Therefore, GPX4 activity and the availability of its GSH substrate provide a critical regulatory checkpoint between reversible oxidative stress and ferroptotic commitment. Importantly, ferroptosis sensitivity is heterogeneous and depends on iron metabolism, lipid composition, antioxidant capacity, and cellular state rather than on a single ROS or iron concentration (125).

This context also explains why a universal pH, ROS concentration, or intracellular iron threshold separating ferroptosis from apoptosis cannot currently be defined. Ferroptosis requires iron-dependent membrane lipid peroxidation, whereas apoptosis is more strongly associated with mitochondrial injury and activation of the intrinsic death machinery. Thus, increasing total ROS does not automatically direct cells toward ferroptosis. The localization and chemical nature of oxidative damage, the availability of peroxidizable membrane lipids, and the effectiveness of ferroptosis-defense systems are equally important. When mitochondrial damage predominates, Bcl-2 family regulation, mitochondrial outer-membrane permeabilization, cytochrome c release, and downstream caspase activation can instead favor apoptotic execution (126).

A mechanistic bridge between MNP-induced ferroptotic stress and mitochondrial apoptosis can emerge when lipid peroxidation extends beyond the plasma membrane and affects mitochondrial phospholipids. MNP-driven iron release and Fenton chemistry can increase lipid ROS, particularly when the SLC7A11/GSH/GPX4 defense axis is impaired. Progressive oxidation of mitochondrial lipids, including cardiolipin, can destabilize mitochondrial membrane organization and facilitate cytochrome c mobilization. Experimental evidence indicates that ferroptotic stimuli can alter cardiolipin-associated membrane permeability and promote cytochrome c release, thereby establishing a direct molecular connection between lipid peroxidation and apoptotic signaling (127). Once mitochondrial stress exceeds the buffering capacity of the cell, activation and oligomerization of BAX and BAK can induce mitochondrial outer membrane permeabilization. Cytochrome c released into the cytosol then promotes Apaf-1 apoptosome formation, followed by caspase-9 and caspase-3/7 activation (128).

This relationship is nevertheless context-dependent rather than obligatory. MOMP and caspase activation are not required for canonical ferroptosis. Instead, they can arise as secondary events when sustained lipid ROS and mitochondrial oxidative injury reach a sufficient level. Regulatory cross-talk can also occur upstream. Bim/Bax signaling has been associated with suppression of SLC7A11 and GPX4, which can increase ferroptotic susceptibility, whereas activation of the apoptotic caspase machinery may alter ACSL4-dependent lipid remodeling and thereby modify ferroptotic sensitivity (129). Thus, MNP-induced oxidative stress can produce a continuum in which GPX4 failure and lipid ROS accumulation initiate ferroptotic pressure, while mitochondrial lipid damage and BAX/BAK-dependent MOMP recruit the classical apoptotic pathway. As illustrated in **Figure 6**, MNP-induced oxidative stress can connect ferroptotic lipid damage with the classical mitochondrial apoptotic pathway through a context-dependent sequence. Suppression of the SLC7A11/GSH/GPX4 defense system promotes lipid ROS accumulation and membrane phospholipid oxidation. When oxidative damage extends to mitochondrial phospholipids, mitochondrial dysfunction and BAX/BAK-dependent MOMP can facilitate cytochrome c release and subsequent activation of the Apaf-1/caspase cascade. This pathway provides a mechanistic explanation for how sustained ferroptotic stress may recruit secondary apoptotic signaling without implying that MOMP is an obligatory component of canonical ferroptosis.

**Figure 6:**
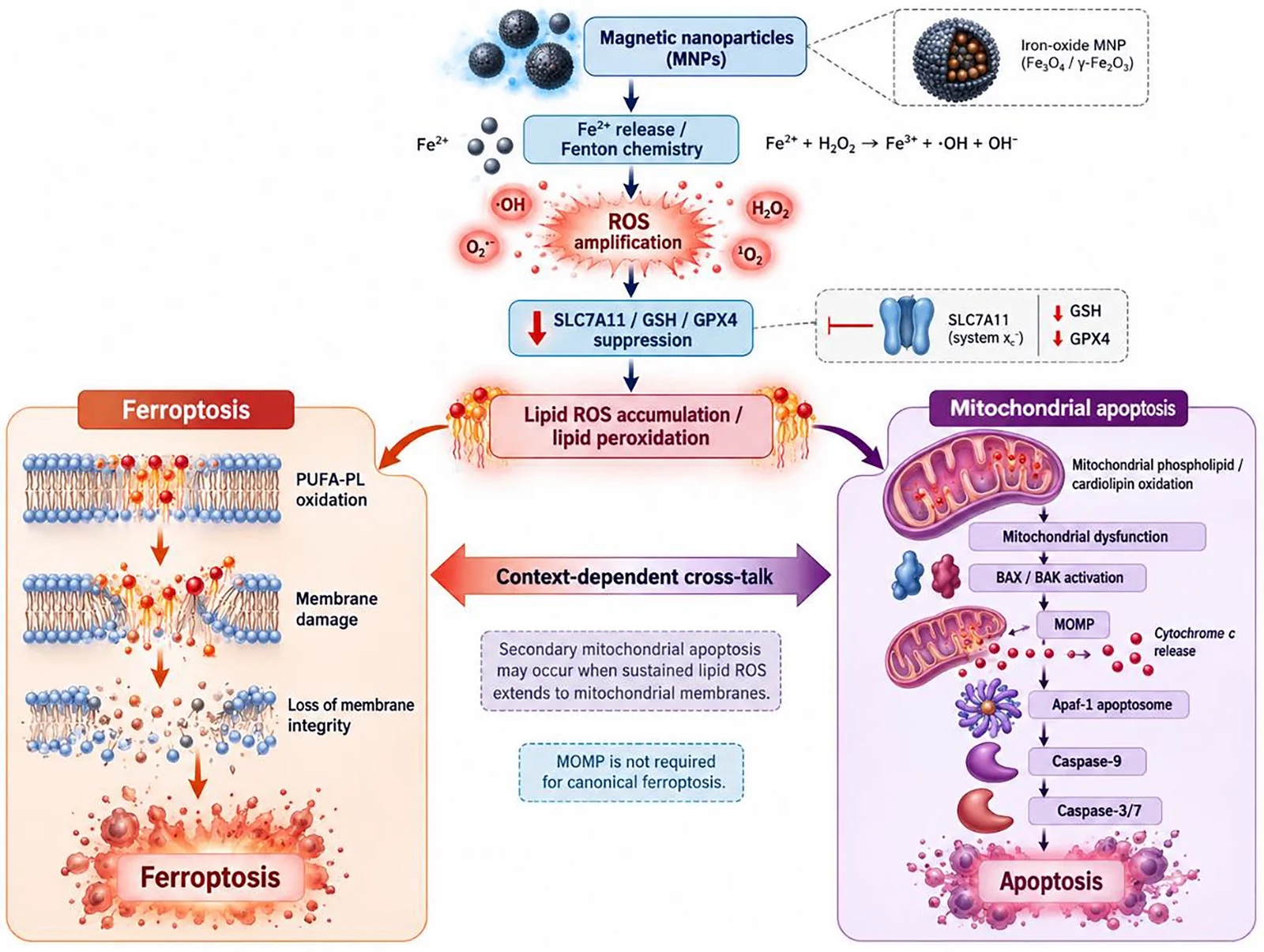
Mechanistic cross-talk between MNP-induced lipid peroxidation, ferroptosis, and mitochondrial apoptosis.

These pathways can also operate simultaneously rather than competitively. A magnetic nanoplatform may enhance Fenton chemistry, disrupt the SLC7A11/GSH/GPX4 axis, and induce mitochondrial oxidative injury within the same tumor cell population. The resulting ferroptotic or apoptotic stress can subsequently influence antitumor immunity. When cell stress or death is accompanied by appropriate immunogenic signals, the exposure or release of danger-associated molecular patterns (DAMPs) can promote antigen-presenting cell activation and subsequent adaptive immune responses. Therefore, immune activation should be considered a downstream and context-dependent consequence of ROS-mediated cell injury rather than a completely independent mechanism (130). As summarized in **Table 3**, the transition from CDT-driven ROS generation to ferroptosis, apoptosis, or immune activation is governed by distinct but interconnected regulatory factors. These include iron-dependent lipid peroxidation, antioxidant defense capacity, mitochondrial susceptibility, and the biochemical conditions of the tumor microenvironment.

**Table 3:** Mechanistic cross-talk and determinants of ROS-mediated cell-death pathways in magnetic nanoplatform-based cancer therapy.

Pathway/transition	Dominant biochemical context	Key regulatory nodes	Mechanistic interpretation	Ref.
CDT → ferroptosis	Increased catalytic metal availability, H_2_O_2_ supply, acidic TME, GSH consumption	Fe²^+^/Fe³^+^ cycling, GSH, lipid peroxidation	Enhanced Fenton chemistry increases ROS production, while depletion of antioxidant capacity favors accumulation of lipid peroxides and ferroptotic damage.	(131)
ROS amplification → ferroptosis	Increased H_2_O_2_ production, lower intracellular pH, GSH depletion, sustained oxidative stress	H_2_O_2_, GSH, iron-dependent lipid oxidation	Simultaneous H_2_O_2_ generation and antioxidant disruption can shift ROS stress toward ferroptosis when lipid peroxide detoxification becomes insufficient.	(132)
ROS → ferroptosis/apoptosis coexistence	Strong oxidative stress affecting both membrane lipids and mitochondria	Lipid peroxidation, mitochondrial injury, apoptosis-associated signaling	Ferroptotic and apoptotic pathways are not mutually exclusive. Multifunctional nanoplatforms can activate several regulated cell-death programs within the same therapeutic system.	(133)
ROS/CDT → immune activation	Oxidative tumor damage combined with magnetic treatment and modulation of the TME	ROS, oxidative stress, immune activation signals	Magnetic nanoplatform-induced oxidative stress can couple direct tumor damage with immune-related therapeutic responses, supporting mechanistic links between catalytic therapy and antitumor immunity.	(124)

## 4 Engineering strategies to enhance therapeutic efficacy

Although MNPs possess intrinsic ROS-generating capabilities, their therapeutic performance is strongly influenced by physicochemical characteristics and biological interactions. Factors such as circulation stability, tumor accumulation, catalytic efficiency, and cellular uptake can significantly affect treatment outcomes. Therefore, considerable efforts have been directed toward engineering magnetic nanoplatforms with improved functionality and selectivity. Recent advances have focused on surface modification, compositional optimization, targeted delivery, and stimuli-responsive designs to enhance ROS generation and maximize antitumor efficacy. These strategies not only improve catalytic performance but also facilitate more precise interactions with the tumor microenvironment. The following sections summarize the major engineering approaches that have been developed to optimize magnetic nanoparticle-based cancer therapy.

### 4.1 Surface engineering and functionalization

Surface engineering is one of the most effective ways to improve the biological performance of MNPs because the outer layer largely determines how the particle behaves in circulation, at the tumor site, and inside cells. Even when two nanoparticles share the same magnetic core, differences in surface chemistry can lead to distinct outcomes in terms of dispersion, stability, cellular interaction, and catalytic accessibility. For this reason, functionalization is not only a formulation step but a key determinant of therapeutic success (134, 135). A major goal of surface modification is to improve colloidal stability in physiological media. Bare MNPs tend to aggregate because of magnetic attraction and high surface energy, which can reduce catalytic efficiency and limit reproducibility (136, 137). Coatings such as polymers, silica, lipids, and biocompatible polysaccharides help maintain a stable dispersion and preserve the active surface area available for redox reactions. At the same time, these coatings can reduce undesired adsorption of serum proteins and minimize premature recognition by the mononuclear phagocyte system (138, 139).

Functional shells also provide a versatile platform for introducing chemical groups that support further modification. Reactive handles on the particle surface make it possible to attach targeting ligands, therapeutic cargos, or tumor-responsive moieties. This modularity is especially useful in cancer nanomedicine, where a single platform may need to combine prolonged circulation, selective tumor recognition, and efficient intracellular delivery. In many cases, the choice of coating directly influences the degree of nanoparticle uptake by cancer cells and can also affect subcellular localization, which is important for ROS-driven mechanisms (140). Another important aspect of surface design is stimulus responsiveness. Some coatings are engineered to remain stable in blood circulation but degrade or rearrange when exposed to acidic pH, elevated glutathione, or specific enzymes in the tumor environment. This kind of selective activation helps expose the catalytic core only after the nanoparticle reaches the diseased site, thereby improving specificity and limiting oxidative damage to healthy tissues. In addition, responsive outer layers can be used to release therapeutic agents in parallel with ROS generation, creating a more coordinated anticancer effect (137, 141).

Overall, surface functionalization allows MNPs to move beyond simple catalytic materials and toward intelligent therapeutic systems. By controlling stability, targeting, and environmental responsiveness, surface engineering provides the foundation for more efficient and selective ROS-mediated cancer treatment.

### 4.2 Composition and structural optimization

The chemical composition of MNPs is a fundamental determinant of their catalytic activity, magnetic behavior, and therapeutic functionality. Even subtle modifications in elemental composition can alter electron-transfer kinetics, redox potential, and ROS-generating efficiency, thereby influencing overall anticancer performance (23). One widely adopted strategy involves incorporating transition metals such as manganese, copper, cobalt, or zinc into iron-based magnetic nanostructures. These dopants can modify the electronic environment of catalytic centers, accelerate redox cycling, and improve Fenton-like activity under physiological conditions (90). Hybrid magnetic nanomaterials have also received increasing attention because they combine complementary physicochemical properties within a single architecture. Integrating magnetic cores with metal oxides, carbon-based materials, or metal–organic frameworks can improve catalytic efficiency while simultaneously enhancing drug-loading capacity, structural stability, or imaging performance (20, 142). Structural engineering provides another effective route for optimizing therapeutic performance. Core–shell, hollow, mesoporous, and yolk–shell architectures create additional active interfaces, shorten diffusion pathways, and improve accessibility of reactants to catalytic sites, resulting in more efficient ROS production (143). Crystal structure and defect engineering further influence catalytic behavior by regulating surface atomic arrangement and the density of active sites. Introducing lattice defects, oxygen vacancies, or disordered regions can facilitate electron transport and promote catalytic reactions without altering the overall nanoparticle composition (144).

Another important consideration is balancing catalytic activity with magnetic performance. Excessive compositional modification may enhance ROS generation but simultaneously weaken magnetic responsiveness, whereas preserving suitable magnetic characteristics is essential for magnetic targeting, imaging, and hyperthermia-based applications. Consequently, optimization requires careful coordination between catalytic efficiency and magnetic functionality (145, 146). Structural and compositional engineering play pivotal roles in regulating catalytic activity, ROS generation efficiency, and therapeutic performance of magnetic nanoplatforms. As summarized in **Table 4**, modifications in metal composition, porosity, active-site density, and drug-loading capacity markedly influence Fenton catalytic efficiency, glutathione depletion, and antitumor efficacy across different magnetic systems.

**Table 4:** Representative magnetic nanoplatforms with optimized compositions and structural architectures for enhanced ROS generation and cancer therapy.

Magnetic nanoplatform	Size/shape	Catalytic efficiency/kinetic parameter	Therapeutic mode	Experimental model	Key outcome	Ref.
Fe-Cu bimetallic MOF/DOX	Spindle-shaped; ~3.5 μm long axis × ~2 μm short axis; mesopores 6.69 nm	40-fold increase in TMB signal; 60% GSH depletion; specific k-value not reported	Chemo-CDT	HeLa cells; no *in vivo* model reported	Cell viability reduced to 35% under 100 μM H_2_O_2_; DOX loading 68.2 ± 2.1%	(147)
Fe-MOF@FU nanoblocks	Concave octahedral; ~500 nm	Vmax = 4.58 × 10^-7^ M s^-^¹; Kcat = 1.83 × 10^-^³ s^-^¹	Chemo-CDT	4T1 cells; no *in vivo* model reported	Cell viability reduced to 17.8%; 76.5% 5-FU release at pH 5.0; GSH depletion	(148)
Fe_3_O_4_@GL-PEG-5-FU nanoparticles	Fe_3_O_4_ particles 15–30 nm; carrier microspheres 0.2–1.5 μm	Specific catalytic k-value not reported	Magnetically responsive/ROS-responsive chemotherapy	MCF-7, MDA-MB-231, and A549 cells	IC_50_: 10.5, 55.8, and 78.1 μg mL^-^¹, respectively	(149)
Hollow Co_3_O_4_@Fe_2_O_3_	Hollow core-shell; crystallite sizes ~21 nm Co_3_O_4_ and ~22 nm Fe_2_O_3_	Degradation rate constant = 0.43 min^-^¹ at pH 7.5	Catalytic Fenton-like model; not evaluated as an anticancer therapy in the cited study	No cancer animal model	Efficient sulfate-radical generation under neutral conditions	(150)

Recent developments increasingly rely on computational materials design and high-throughput screening to predict optimal compositions before experimental synthesis. These approaches accelerate the discovery of multifunctional magnetic nanomaterials with improved catalytic performance, structural stability, and biological compatibility, thereby supporting the development of more efficient ROS-mediated cancer therapeutics.

### 4.3 Targeted delivery strategies

Efficient delivery of MNPs to tumor tissues is a prerequisite for maximizing therapeutic efficacy while minimizing systemic toxicity, making targeting strategies a central component of nanomedicine design (151). Passive targeting primarily relies on the enhanced permeability and retention (EPR) effect, whereby nanosized materials preferentially accumulate within tumors owing to abnormal vascular permeability and impaired lymphatic drainage. Although the magnitude of the EPR effect varies among tumor types and patients, it remains an important mechanism for nanoparticle localization in many preclinical studies (152, 153). To overcome the limitations of passive accumulation, active targeting strategies have been developed by decorating nanoparticle surfaces with ligands capable of recognizing overexpressed receptors on cancer cells or tumor-associated stromal cells. These ligands include antibodies, peptides, aptamers, carbohydrates, and small molecules that promote receptor-mediated internalization and improve cellular specificity (154).

Among the various targeting ligands, folic acid has been extensively investigated because folate receptors are highly expressed in numerous solid tumors while exhibiting relatively low expression in most normal tissues. Functionalization with folic acid has been shown to enhance selective nanoparticle uptake and improve therapeutic performance in multiple experimental cancer models (155). Magnetic targeting represents a distinctive advantage of MNPs over many other nanocarriers. Application of an external magnetic field can increase nanoparticle retention at the tumor site, reduce off-target distribution, and improve local therapeutic concentration without altering nanoparticle composition (156).

Biomimetic delivery systems have recently emerged as an innovative strategy for improving tumor homing. Coating MNPs with cell membranes derived from erythrocytes, platelets, macrophages, or cancer cells allows the nanoplatforms to evade immune clearance while exploiting natural biological interactions to improve tumor accumulation (157, 158).

Another promising approach involves microenvironment-responsive targeting, in which nanoparticles remain relatively inactive during circulation. However, undergo structural or chemical changes after encountering tumor-associated stimuli such as acidic pH, overexpressed enzymes, hypoxia, or elevated glutathione concentrations. These responsive systems improve therapeutic selectivity by restricting nanoparticle activation to diseased tissues (18, 159).

Overall, advances in targeted delivery have transformed MNPs from passive carriers into highly specialized therapeutic systems capable of achieving greater precision, improved tumor selectivity, and enhanced ROS-mediated anticancer activity.

### 4.4 Stimuli-responsive magnetic nanoplatforms

Stimuli-responsive magnetic nanoplatforms are designed to undergo physicochemical or functional changes only after encountering specific internal or external triggers, thereby improving therapeutic precision and reducing premature activation (160). Among endogenous stimuli, acidic pH is one of the most frequently exploited signals because the extracellular environment of many solid tumors is more acidic than that of healthy tissues, allowing pH-sensitive nanocarriers to selectively expose catalytic sites or release therapeutic payloads (161).

Redox-responsive systems represent another important class of smart nanoplatforms, where elevated intracellular glutathione concentrations promote cleavage of redox-sensitive linkages, facilitating controlled activation or cargo release after cellular internalization (162). Enzyme-responsive MNPs have also attracted increasing attention because tumor-associated enzymes, including matrix metalloproteinases and hyaluronidases, can selectively degrade engineered coatings and trigger localized therapeutic responses (163). Hypoxia-responsive nanoplatforms exploit oxygen-deficient regions within solid tumors by incorporating chemical groups that undergo structural transformation under low-oxygen conditions, thereby improving treatment efficiency in poorly vascularized lesions (164).

External magnetic fields provide an additional level of control by guiding MNPs toward target tissues or activating therapeutic functions remotely without direct physical contact with the tumor (165). Other exogenous triggers, including near-infrared light, ultrasound, and alternating magnetic fields, have been integrated into multifunctional magnetic nanoplatforms to achieve spatially and temporally controlled therapeutic activation while minimizing exposure of healthy tissues (166). The combination of multiple responsive mechanisms within a single nanosystem has emerged as a promising strategy because dual- or multi-stimuli-responsive platforms can adapt to the heterogeneous characteristics of tumors and produce more reliable therapeutic outcomes than single-trigger systems (160). Beyond therapeutic activation, recent advances have expanded stimuli-responsive nanoplatforms toward multifunctional nanozyme systems capable of integrating responsive behavior with biosensing and real-time biomedical evaluation. Graphene quantum dot/hydrogel nanozyme composites represent an emerging class of intelligent nanomaterials that combine catalytic activity, optical responsiveness, and diagnostic capability, highlighting the growing convergence between responsive nanomedicine and next-generation theranostic platforms (167).

Zhang et al. developed mesoporous magnetic nanocarriers containing disulfide bonds (CHO-SMNPs) capable of responding simultaneously to acidic pH, intracellular glutathione, and NIR irradiation for controlled drug release and synergistic therapy (**Figure 7a**). Drug release experiments demonstrated that acidic conditions (pH 5.5) and elevated GSH concentrations significantly accelerated DOX release compared with physiological conditions (**Figures 7b-i**). Furthermore, NIR irradiation further enhanced drug liberation through photothermal activation of the Fe_3_O_4_ core (**Figures 7b-ii**). The therapeutic efficacy of the triple-responsive nanoplatform was further verified by live/dead staining assays. Compared with monotherapy groups, the combined chemo-photothermal treatment induced substantially higher levels of tumor cell death, indicating a strong synergistic therapeutic effect between chemotherapy and photothermal therapy (**Figure 7c**). In addition, infrared thermal imaging confirmed the excellent photothermal conversion capability of CHO-SMNPs, where increasing nanoparticle concentration resulted in progressively higher temperature elevations under 808 nm laser irradiation (**Figure 7d**). These results demonstrated the suitability of Fe_3_O_4_-containing nanocarriers for NIR-triggered therapeutic applications (168). Jia et al. reported pH-responsive nanoclusters assembled from ultrasmall IONPs (USIO NCs) for tumor microenvironment-responsive theranostics. TEM analysis confirmed the ultrasmall and well-dispersed morphology of the iron oxide nanoclusters (**Figure 7e**). Furthermore, fluorescence emission studies revealed significant fluorescence recovery under acidic conditions (pH 5.5) compared with physiological pH (7.4), confirming dissociation of the nanoclusters and the associated release of encapsulated molecules in the tumor microenvironment (**Figure 7f**) (169).

**Figure 7:**
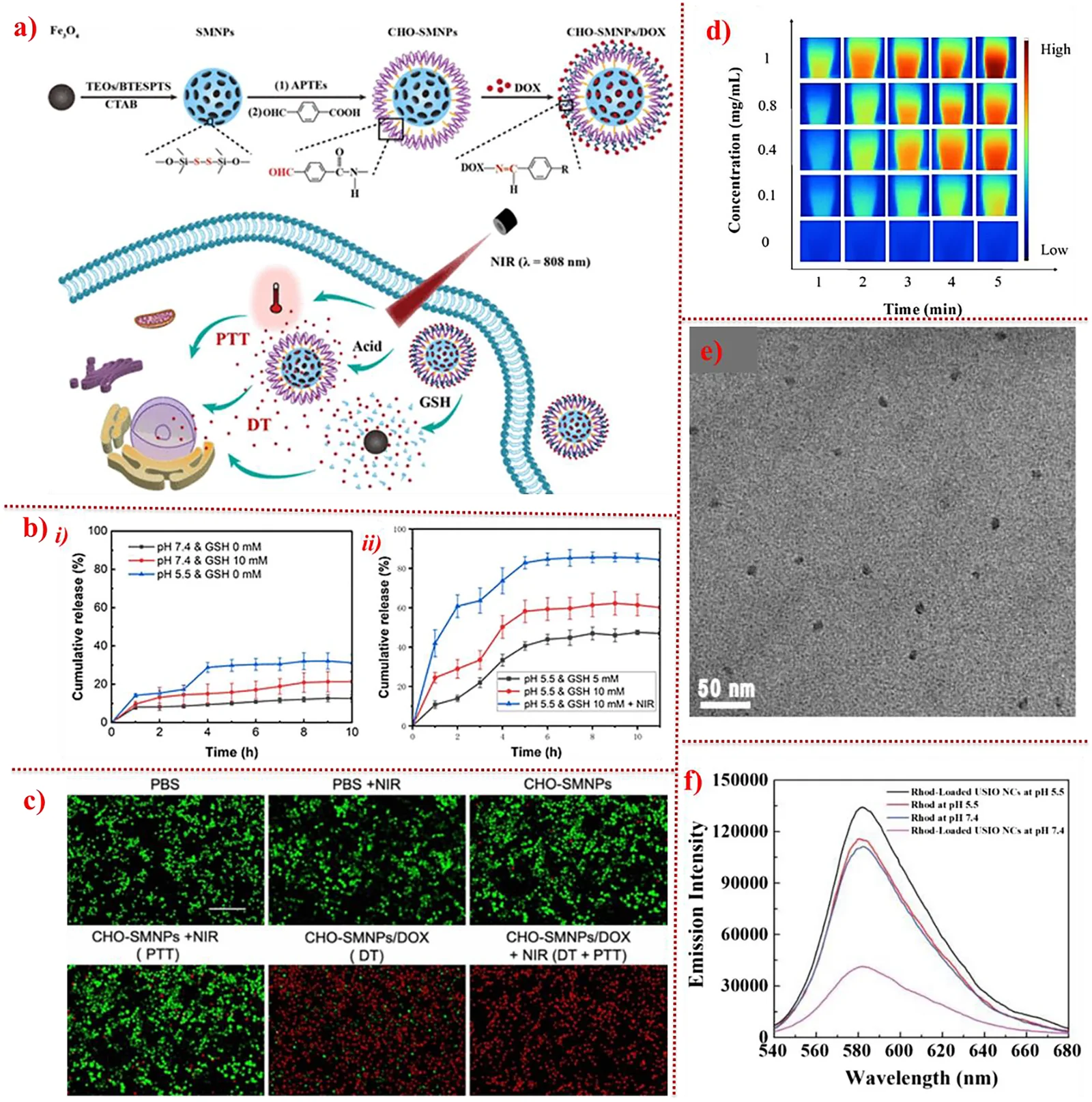
Experimental evidence demonstrating stimuli-responsive magnetic nanoplatforms for controlled drug delivery and cancer therapy. **(a)** Schematic illustration of the pH-, GSH-, and NIR-responsive magnetic nanocarrier (CHO-SMNPs/DOX) and its intracellular drug release behavior under tumor microenvironment conditions. **(b-i)** Cumulative DOX release profiles from CHO-SMNPs/DOX under different pH and GSH concentrations. **(b-ii)** Effect of NIR irradiation on drug release behavior under acidic and reductive conditions. **(c)** Live/dead fluorescence staining images showing therapeutic outcomes after PBS, PTT, DT, and combined chemo-photothermal treatment (DT + PTT). d) Infrared thermal images of CHO-SMNP dispersions at different concentrations under 808 nm laser irradiation. Reprinted with permission from ref (168). **(e)** TEM image of ultrasmall iron oxide nanoclusters (USIO NCs). **(f)** Fluorescence emission spectra of Rhod-loaded USIO nanoclusters under physiological and acidic conditions, demonstrating pH-responsive dissociation behavior. Reprinted with permission from ref (169). copyright *^©^ 2020 Elsevier Ltd. All rights reserved. License Number: 6300560980201*.

Collectively, stimuli-responsive engineering has transformed MNPs into intelligent therapeutic platforms capable of responding dynamically to biological and physical cues, representing an important step toward precision nanomedicine for ROS-mediated cancer therapy.

## 5 Combination therapeutic approaches

While catalytic ROS generation can effectively suppress tumor growth, a single therapeutic modality is often insufficient to eliminate heterogeneous tumors or prevent disease recurrence. Integrating magnetic nanoparticle-mediated ROS production with complementary treatment strategies has therefore become an important direction in contemporary cancer nanomedicine, offering opportunities to improve efficacy through cooperative biological mechanisms. The rationale for combination therapy extends beyond simply increasing tumor cell damage. Distinct therapeutic modalities frequently target different molecular pathways, allowing one treatment to compensate for the limitations of another and reducing the likelihood of therapeutic resistance. MNPs provide a versatile platform for multimodal treatment because their physicochemical properties enable the simultaneous incorporation of catalytic, magnetic, imaging, and drug-delivery functions within a single nanosystem. This multifunctionality facilitates coordinated therapeutic interventions without requiring multiple independent nanocarriers. Careful integration of complementary therapies can also reduce the required dose of individual treatments, thereby limiting systemic toxicity while maintaining or even enhancing overall antitumor activity. Such synergistic interactions have become a major objective in the development of next-generation magnetic nanoplatforms. The following sections summarize the principal combination strategies involving MNPs, including their integration with chemodynamic therapy, magnetic hyperthermia, photothermal therapy, photodynamic therapy, chemotherapy, and immunotherapy, highlighting how these approaches collectively improve ROS-mediated cancer treatment.

### 5.1 Magnetic nanoparticle-mediated chemodynamic therapy

CDT has become one of the most representative applications of MNPs because iron-containing nanomaterials can continuously catalyse endogenous hydrogen peroxide into highly reactive hydroxyl radicals within tumor tissues, providing localized oxidative damage without external energy input (38, 170). Compared with conventional catalytic systems, MNPs provide sustained catalytic activity by serving as reservoirs of redox-active metal ions that gradually participate in intracellular oxidation–reduction reactions following nanoparticle degradation (171). The effectiveness of magnetic CDT is strongly influenced by the biochemical composition of the tumor microenvironment, particularly hydrogen peroxide concentration, local acidity, and intracellular antioxidant levels, all of which regulate catalytic efficiency (131).

Several engineering strategies have therefore been proposed to overcome substrate limitations by increasing endogenous hydrogen peroxide production or simultaneously consuming intracellular glutathione to preserve ROS activity after formation (172). Another promising approach involves incorporating secondary catalytic metals, including manganese, copper, or cobalt, into iron-based magnetic nanoplatforms to strengthen Fenton-like reactions and expand catalytic performance across broader physiological conditions (55). Mesoporous and hollow magnetic nanostructures have also demonstrated improved CDT efficiency because their enlarged internal surface area facilitates substrate diffusion and exposes a greater number of catalytically active sites (173). Recent studies have further demonstrated that integrating CDT with drug delivery functions enables synchronized ROS generation and controlled therapeutic release from the same nanoplatform, thereby improving treatment efficiency while reducing unnecessary exposure of healthy tissues (82).

Collectively, these developments demonstrate that magnetic nanoparticle-mediated CDT has evolved from a simple catalytic concept into an advanced multifunctional therapeutic strategy capable of addressing multiple biological barriers encountered during cancer treatment.

### 5.2 Magnetic hyperthermia-assisted therapy

Magnetic hyperthermia (MHT) is an established therapeutic technique in which MNPs convert electromagnetic energy from an alternating magnetic field into localized heat, producing controlled temperature elevation within tumor tissues. This localized heating can directly impair cancer cell survival while minimizing thermal damage to surrounding healthy structures (174). The heating efficiency of MNPs depends on intrinsic properties such as particle size, magnetic anisotropy, crystal structure, and saturation magnetization, all of which influence energy dissipation through Néel and Brownian relaxation mechanisms (175, 176).

Moderate hyperthermia can destabilize cellular membranes, denature intracellular proteins, and interfere with DNA repair pathways, thereby increasing the susceptibility of tumor cells to oxidative injury (177). Heat generated during MHT has also been reported to accelerate intracellular catalytic reactions and enhance ROS accumulation, producing stronger oxidative damage than catalytic therapy alone (73, 124). Another important consequence of localized heating is improved tumor perfusion and vascular permeability, which can facilitate deeper nanoparticle penetration and increase the accessibility of therapeutic agents within solid tumors (178).

Recent nanoplatforms have integrated magnetic hyperthermia with catalytic nanomedicine by combining heat generation and ROS production within a single magnetic architecture, allowing simultaneous thermal and oxidative stress to attack multiple cellular targets (82, 179). Precise regulation of treatment temperature remains essential because excessive heating may damage adjacent tissues, whereas insufficient thermal exposure may fail to achieve meaningful therapeutic enhancement (180, 181). Consequently, optimization of magnetic field parameters and nanoparticle composition continues to be a major focus of current research.

Overall, magnetic hyperthermia provides a valuable complementary strategy for ROS-mediated cancer therapy by improving catalytic efficiency, sensitizing tumor cells to oxidative injury, and expanding the therapeutic capabilities of multifunctional magnetic nanoplatforms.

### 5.3 Photothermal and photodynamic synergistic therapy

Phototherapy-based strategies have attracted considerable attention in cancer nanomedicine because they enable spatiotemporal control over therapeutic activation through external light irradiation. Among these approaches, photothermal therapy (PTT) and photodynamic therapy (PDT) are particularly attractive due to their complementary mechanisms of action and their compatibility with multifunctional nanoplatforms (182).

PTT relies on the conversion of absorbed light energy into heat by photothermal agents following irradiation, most commonly within the near-infrared (NIR) region where tissue penetration is relatively high and damage to surrounding tissues is reduced (183). Local temperature elevation can induce protein denaturation, membrane destabilization, cytoskeletal disruption, and metabolic dysfunction, ultimately compromising tumor viability (184). Unlike PTT, PDT is mediated by photosensitizers that transfer absorbed photon energy to nearby molecular oxygen, generating cytotoxic species such as singlet oxygen (^1O_2_), superoxide radicals (O_2_•^-^), and hydroxyl radicals (•OH). These reactive intermediates attack cellular structures and initiate oxidative injury in illuminated regions (185). Despite their effectiveness, both modalities possess intrinsic limitations when applied individually. Hyperthermia-based treatments may exhibit incomplete tumor eradication due to heterogeneous heat distribution, whereas PDT frequently suffers from oxygen dependence and reduced efficacy in hypoxic tumors (186, 187). The integration of MNPs into phototherapeutic systems offers opportunities to overcome several of these limitations simultaneously. Magnetic nanostructures can act as carriers for photosensitizers, support magnetic targeting, improve tumor accumulation, and provide additional catalytic functions associated with ROS generation (188, 189).

One important advantage of PTT-assisted catalytic therapy is the temperature dependence of chemical reaction kinetics. Elevated local temperatures can accelerate electron-transfer processes and increase the reaction rate of catalytic systems, thereby strengthening ROS generation and oxidative injury within malignant tissues (190). Thermal exposure can also increase membrane fluidity and permeability, facilitating nanoparticle internalization and improving intracellular access to therapeutic targets. Enhanced transport across biological barriers may further increase the effectiveness of catalytic and photodynamic processes (191). Conversely, oxidative stress generated during PDT may sensitize tumor cells to subsequent thermal treatment by disrupting antioxidant defense systems and reducing the ability of cells to repair heat-induced damage. This bidirectional interaction contributes to stronger biological responses than either modality alone (192, 193).

Recent nanoplatforms increasingly combine PTT, PDT, CDT, and magnetic functionalities within a single architecture to achieve multimodal therapy. Such systems can simultaneously generate heat, release catalytic metal ions, produce ROS, and improve tumor localization through magnetic guidance, thereby attacking cancer cells through multiple independent mechanisms (194). These multifunctional interactions may activate several regulated cell death pathways, including apoptosis, ferroptosis, and immunogenic cell death, which can reduce treatment resistance and improve long-term therapeutic outcomes (195). Nevertheless, several technical challenges remain, including limited penetration depth of excitation light, heterogeneous photosensitizer distribution, photobleaching, and variability in tumor oxygenation. Addressing these issues will require continued advances in nanomaterial engineering, optical technologies, and tumor-responsive therapeutic design.

Li et al. demonstrated that the combination of PTT and PDT significantly enhanced antitumor efficacy compared with individual treatment modalities. As shown in **Figure 8a**, live/dead staining images revealed a marked increase in dead cells following simultaneous irradiation with both 660 nm and 808 nm lasers, whereas treatment with either PTT or PDT alone resulted in substantially lower levels of cell death. Furthermore, flow cytometric analysis presented in **Figure 8b** confirmed that combined phototherapy induced the highest apoptotic population, indicating a strong synergistic interaction between hyperthermia and ROS-mediated oxidative damage. In addition, confocal fluorescence imaging using DCFH-DA probes demonstrated that intracellular ROS production was dramatically increased under dual-laser irradiation conditions (**Figure 8c**), further validating the enhanced PDT activity achieved in the combined treatment group. These findings indicate that mitochondrial-targeted magnetic nanoheterostructures can simultaneously induce thermal ablation and oxidative stress, leading to superior therapeutic outcomes in triple-negative breast cancer models (196). Similarly, Xue et al. reported that magnetic Prussian blue nanoplatforms loaded with indocyanine green generated substantial intracellular ROS and significantly improved tumor cell killing upon NIR irradiation. As illustrated in **Figure 8d**, fluorescence microscopy images showed a pronounced increase in ROS-associated fluorescence signals after laser activation of FPPI nanoparticles, confirming effective PDT activation alongside photothermal conversion. The integration of magnetic targeting, photothermal heating, and ROS generation enabled efficient synergistic tumor destruction, highlighting the potential of multifunctional magnetic nanoplatforms for combined phototherapy applications (197).

**Figure 8:**
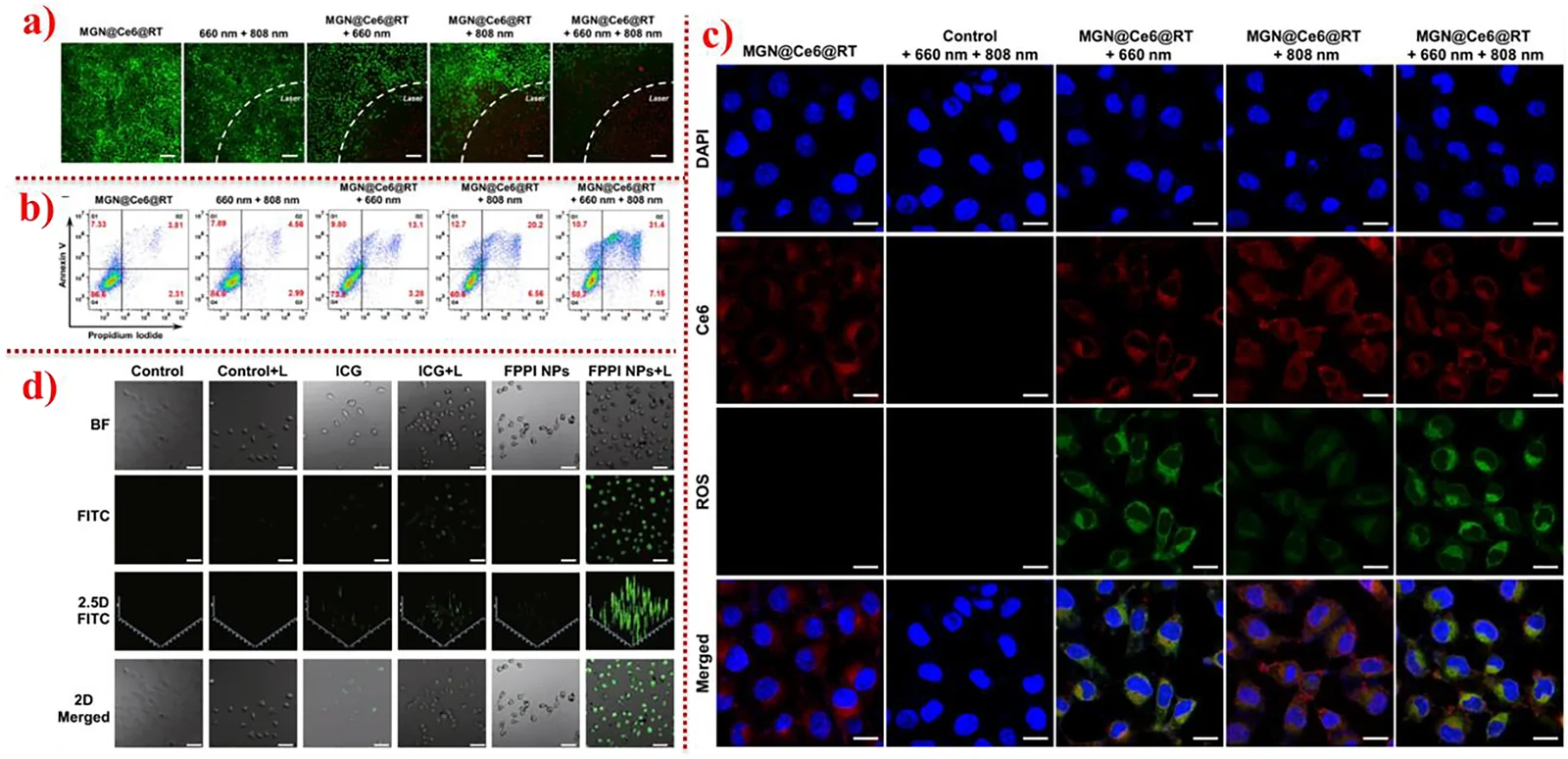
Representative experimental evidence demonstrating the synergistic mechanisms and therapeutic outcomes of combined PTT and PDT. **(a)** Calcein-AM/PI live/dead staining images showing enhanced cancer cell ablation after combined 660 nm and 808 nm laser irradiation compared with individual PTT or PDT treatment. **(b)** Flow cytometric analysis of Annexin V-FITC/PI staining confirming increased apoptosis following combined photothermal-photodynamic treatment. **(c)** Confocal fluorescence imaging of intracellular ROS generation using DCFH-DA probe after exposure to MGN@Ce6@RT under different irradiation conditions, showing the highest ROS production in the combined PTT/PDT group. Reprinted with permission from ref (196). copyright *^©^ 2020 Elsevier B.V. All rights reserved. License Number: 6300570678285.* **(d)** Representative fluorescence microscopy images illustrating intracellular ROS generation and cell killing effects induced by FPPI nanoparticles under NIR irradiation. Reprinted with permission from ref (197).

Overall, the experimental evidence presented in **Figure 8** clearly demonstrates that the integration of PTT and PDT within a single magnetic nanoplatform results in enhanced ROS generation, increased apoptosis, and superior cancer cell eradication compared with monotherapy approaches, thereby supporting the growing interest in multimodal phototherapeutic strategies for cancer treatment.

### 5.4 Combination with chemotherapy

The integration of chemotherapy with ROS-generating MNPs has attracted considerable interest as a strategy to overcome the intrinsic limitations of conventional anticancer drugs, including multidrug resistance, poor tumor selectivity, dose-dependent toxicity, and inadequate intracellular accumulation. The ability of magnetic nanoplatforms to simultaneously deliver chemotherapeutic agents and generate oxidative stress provides an opportunity to achieve synergistic antitumor activity while reducing systemic side effects (198, 199). One of the major advantages of this combination arises from the influence of oxidative stress on cellular defense pathways. Elevated ROS levels can impair antioxidant systems, disrupt DNA repair mechanisms, and weaken drug-resistance networks, thereby increasing the sensitivity of tumor cells to chemotherapeutic agents. This effect is particularly relevant in resistant tumors that exhibit enhanced detoxification capacity or increased drug efflux activity (200, 201). Several commonly used anticancer drugs demonstrate improved efficacy in oxidative environments. Doxorubicin, for example, exerts its activity through DNA intercalation and topoisomerase inhibition, while additional ROS generation can intensify DNA strand damage and accelerate cell death. Similarly, cisplatin-mediated DNA crosslinking becomes more effective when oxidative stress compromises cellular repair pathways (202).

MNPs offer unique opportunities for co-delivery strategies because their surfaces can be engineered to simultaneously accommodate catalytic components and drug molecules. Such systems allow synchronized intracellular drug release and ROS production, increasing local therapeutic concentration while minimizing premature leakage during circulation (140, 203). Drug loading can be achieved through adsorption, covalent conjugation, encapsulation, or incorporation into porous structures surrounding the magnetic core (204). Mesoporous silica shells, polymeric coatings, and metal–organic framework layers have all been investigated as reservoirs capable of carrying substantial quantities of chemotherapeutic agents while preserving catalytic activity (137). Stimuli-responsive release mechanisms further enhance therapeutic precision. Many magnetic nanoplatforms remain relatively stable under physiological conditions but release their payload after exposure to acidic pH, elevated glutathione concentrations, or tumor-associated enzymes. Such selective activation improves tumor specificity and reduces unintended exposure of healthy tissues to cytotoxic drugs (205).

External magnetic guidance represents another important advantage of these systems. Application of a magnetic field can improve local nanoparticle retention and increase intratumoral drug accumulation, thereby enhancing treatment efficacy while lowering systemic drug distribution. This approach may be particularly valuable for highly aggressive tumors that are difficult to treat using conventional systemic chemotherapy alone (206, 207). Recent multifunctional nanoplatforms have integrated drug delivery, catalytic ROS generation, imaging capability, and targeting ligands into a single therapeutic system. These designs allow simultaneous diagnosis, monitoring, and treatment, supporting the growing concept of theranostic nanomedicine (208). Despite encouraging progress, several challenges remain before widespread clinical translation can be achieved. Drug loading efficiency, release reproducibility, manufacturing scalability, and long-term biosafety continue to require careful investigation. Moreover, variations in tumor biology among patients may influence treatment response and should be considered during future nanoplatform design.

Magnetic nanoplatforms have demonstrated remarkable capabilities in improving the precision and efficacy of chemotherapeutic delivery through the integration of magnetic guidance, active targeting ligands, and stimuli-responsive release mechanisms. Liu et al. developed a dual-targeted Fe_3_O_4_@C/ZnO-DOX-FA nanoplatform combining magnetic targeting mediated by Fe_3_O_4_ cores with folic acid receptor-mediated cellular uptake. Magnetization measurements confirmed the preservation of superparamagnetic behavior after surface functionalization, enabling efficient magnetic manipulation without residual magnetization-induced aggregation (**Figure 9a**). Furthermore, cytotoxicity assays showed a concentration-dependent reduction in HeLa cell viability, with the Fe_3_O_4_@C/ZnO-DOX-FA system under laser irradiation exhibiting the highest anticancer efficacy due to synergistic chemo-photothermal effects (**Figure 9b**). Cellular uptake studies using FITC labeling and flow cytometry demonstrated significantly higher intracellular accumulation for folic acid-modified nanoparticles compared with non-functionalized counterparts, confirming the contribution of receptor-mediated endocytosis to enhanced tumor targeting (**Figures 9c–f**) (209). Similarly, Nigam and Bahadur designed dendrimer-functionalized IONPs capable of thermally activated chemotherapy and pH-responsive drug delivery. Their results demonstrated that both PAMAM-coated and peptide dendrimer-coated MNPs exhibited limited doxorubicin leakage under physiological conditions (pH 7.3), whereas acidic environments resembling tumor intracellular compartments (pH 5.0) induced rapid and sustained drug release. Notably, KR2-IO nanoparticles achieved higher cumulative release than PAMAM-IO formulations under acidic conditions, suggesting improved responsiveness of peptide dendrimers to tumor-associated pH gradients (**Figure 9g**). Such behavior minimizes premature systemic drug leakage while maximizing intracellular drug bioavailability within tumor tissues (210).

**Figure 9:**
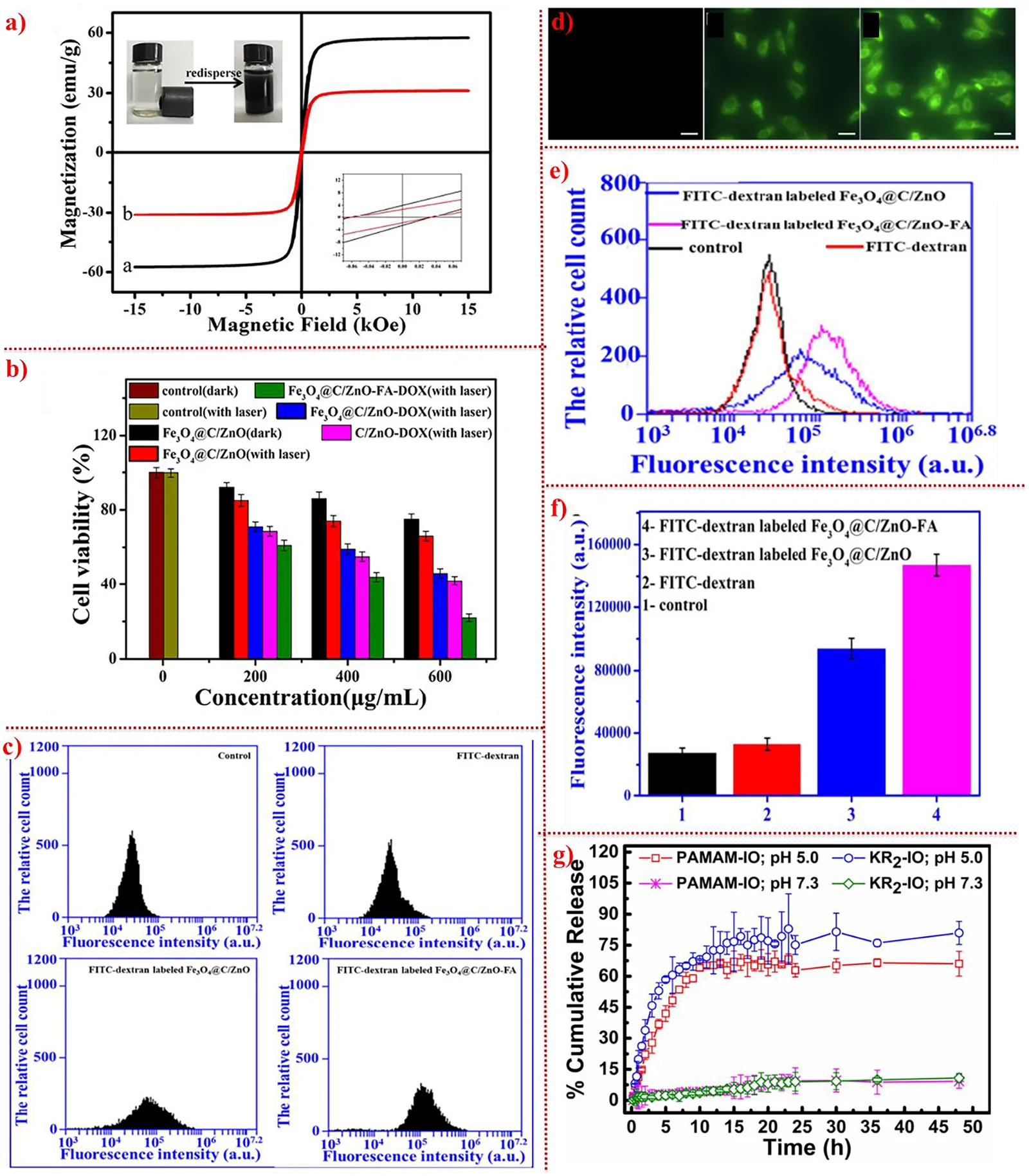
Representative examples of multifunctional magnetic nanoplatforms for targeted and stimuli-responsive cancer chemotherapy. **(a)** Magnetic hysteresis loops and magnetic responsiveness of Fe_3_O_4_@C/ZnO and Fe_3_O_4_@C/ZnO-FA nanoplatforms demonstrating superparamagnetic behavior and efficient magnetic manipulation. **(b)** Cell viability of HeLa cells after treatment with Fe_3_O_4_@C/ZnO-based formulations under different concentrations and laser irradiation conditions, highlighting the enhanced cytotoxicity of combined chemo-photothermal therapy. **(c–f)** Cellular uptake evaluation using FITC-dextran labeling, confocal fluorescence imaging and flow cytometry analysis, revealing significantly enhanced internalization of folic acid-functionalized Fe_3_O_4_@C/ZnO-FA nanoparticles due to folate receptor-mediated endocytosis. Reprinted with permission from ref (209). copyright *^©^ 2020 Elsevier B.V. All rights reserved. License Number: 6300571145087. ***(g)** pH-responsive cumulative release profiles of doxorubicin from PAMAM-IO and KR2-IO nanocarriers under physiological and acidic tumor-like conditions, demonstrating accelerated drug release in acidic environments. Reprinted with permission from ref (210). copyright *^©^ 2017 Elsevier B.V. All rights reserved. License Number: 6300571407414*.

Collectively, these studies highlight that combining magnetic targeting, ligand-mediated active uptake, and tumor-responsive release mechanisms can substantially enhance therapeutic selectivity and anticancer efficacy while reducing off-target toxicity. The integration of these functionalities within a single nanoplatform represents an important strategy toward next-generation precision chemotherapy systems.

### 5.5 Combination with immunotherapy

The combination of magnetic nanoparticle-mediated ROS therapy with immunotherapy has attracted growing attention as a promising strategy for generating durable and systemic antitumor responses. Conventional ROS-mediated therapies primarily focus on direct tumor destruction, whereas immunotherapy aims to stimulate host immune defenses capable of recognizing and eliminating malignant cells throughout the body. The integration of these approaches therefore offers opportunities to couple local cytotoxicity with long-term immune surveillance (211).

One of the most important links between ROS generation and immune activation is immunogenic cell death (ICD). Unlike non-immunogenic forms of cell death, ICD is accompanied by the release or exposure of danger-associated molecular patterns (DAMPs), including calreticulin, ATP, and HMGB1, which function as endogenous alarm signals for the immune system. These molecules facilitate antigen presentation and initiate adaptive immune responses against tumor-associated antigens (212). Dendritic cells represent a central component of this process because they capture tumor-derived antigens and present them to T lymphocytes in secondary lymphoid organs. The activation of cytotoxic CD8+ T cells subsequently enables immune-mediated elimination of residual tumor cells that may remain after primary treatment. This mechanism is particularly important for preventing recurrence and suppressing metastatic dissemination (213, 214).

ROS-mediated therapies can also influence immune-cell composition within tumors by modulating macrophage polarization, enhancing dendritic-cell maturation, and increasing infiltration of effector T lymphocytes. Several studies have demonstrated that oxidative stress can shift macrophages from immunosuppressive M2 phenotypes toward tumoricidal M1 phenotypes, thereby improving the immunological status of the tumor microenvironment (215). Immune checkpoint blockade represents another important application of these synergistic approaches. Antibodies targeting PD-1, PD-L1, and CTLA-4 have revolutionized cancer treatment but often exhibit limited efficacy because of poor tumor immunogenicity and inadequate T-cell activation. ROS-generating magnetic nanoplatforms can enhance antigen release and immune recognition, thereby improving responsiveness to checkpoint inhibition (216).

Magnetic hyperthermia may further strengthen immune activation by promoting local inflammatory responses and increasing antigen release within heated tumor regions. This phenomenon has led to the concept of *in situ* vaccination, in which local treatment stimulates systemic antitumor immunity capable of targeting distant lesions (217). MNPs also offer opportunities for direct delivery of immunotherapeutic agents. Cytokines, immune adjuvants, nucleic acids, and checkpoint inhibitors can be conjugated to magnetic nanocarriers to improve local accumulation and reduce systemic toxicity. Such targeted delivery systems may enhance therapeutic efficacy while minimizing immune-related adverse events frequently associated with systemic immunotherapy administration (211). The incorporation of imaging capabilities provides an additional advantage for these multifunctional systems. Magnetic resonance imaging, fluorescence imaging, and photoacoustic imaging can be integrated into immunotherapeutic nanoplatforms to monitor biodistribution, therapeutic response, and immune activation in real time. This theranostic capability may facilitate personalized treatment optimization in future clinical applications (218, 219).

Despite encouraging progress, several challenges continue to impede clinical translation. Tumor heterogeneity, immune exhaustion, variability in treatment timing, and the complexity of immune signaling networks remain significant barriers to reproducible therapeutic outcomes. In addition, excessive ROS generation may impair immune-cell function if oxidative stress exceeds tolerable limits, emphasizing the importance of careful therapeutic balance. As illustrated in **Figures 10a, b**, Perica et al. demonstrated that exposure of paramagnetic nano-artificial antigen-presenting cells (nano-aAPCs) to an external magnetic field promoted the aggregation of nanoparticle-bound TCR complexes on the T-cell membrane, resulting in significantly larger CD3ϵ clusters compared with non-cognate particles or nano-aAPCs in the absence of magnetic stimulation. The enlargement of receptor clusters was associated with enhanced antigen-specific T-cell activation and improved signaling efficiency (220).

**Figure 10:**
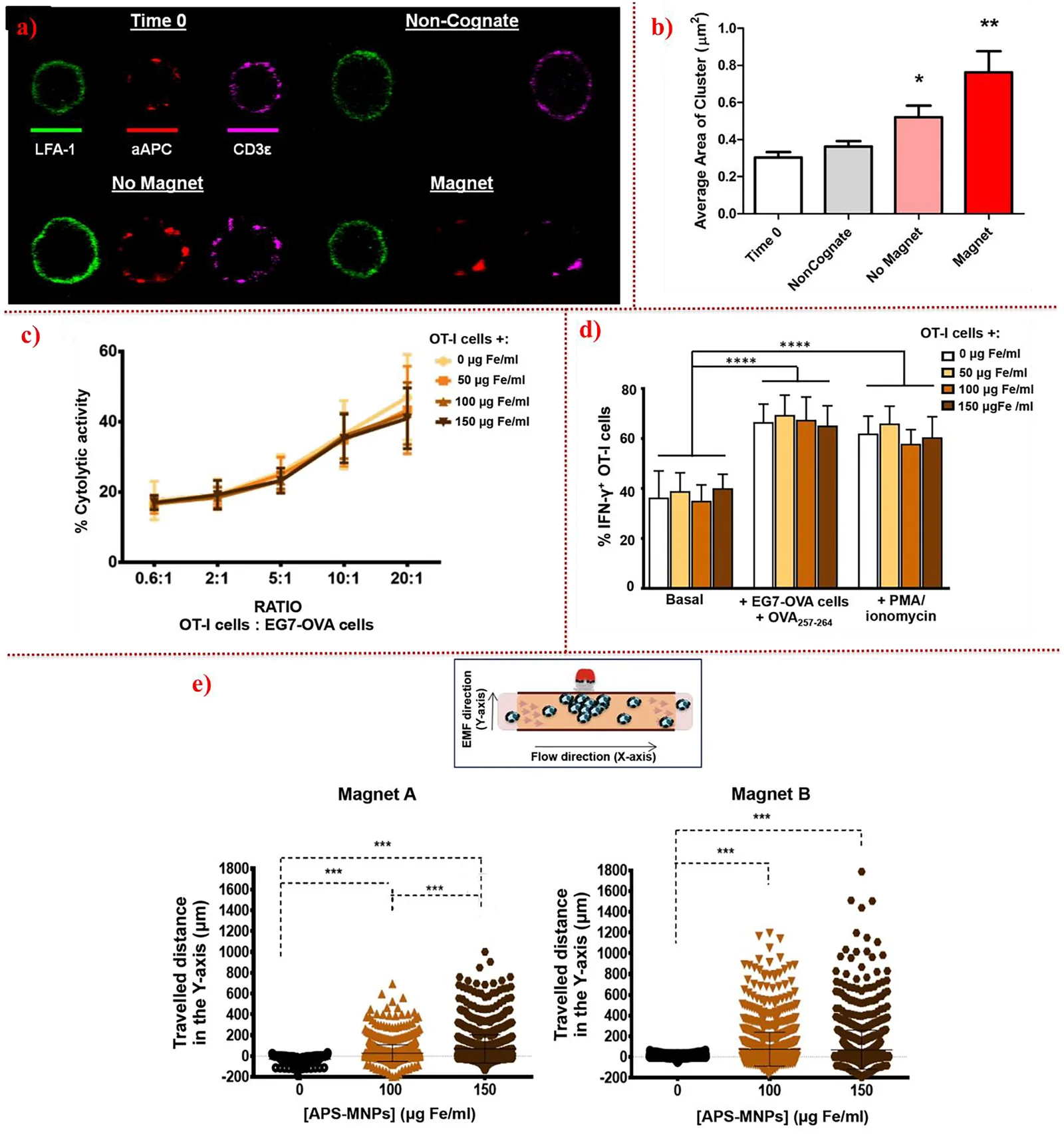
*Magnetic modulation of T-cell activation and trafficking by nanoparticle-based immunotherapy platforms*. **(a, b)** Confocal imaging and quantitative analysis of T-cell receptor (TCR) clustering induced by paramagnetic nano-artificial antigen-presenting cells (nano-aAPCs) under an external magnetic field. Reprinted with permission from ref (220). **(c, d)** Evaluation of the antitumor functions of APS-MNP-loaded OT-I CD8+ T cells. **(e)** Magnetic retention of APS-MNP-loaded OT-I CD8+ T cells under dynamic flow conditions. Reprinted with permission from ref (221).

As shown in **Figures 10c, d**, Sanz-Ortega et al. reported that the association of OT-I CD8+ T cells with APS-coated MNPs did not significantly impair their cytotoxic activity against EG7-OVA target cells or their ability to produce IFN-γ following antigen-specific stimulation, indicating preservation of key effector functions after nanoparticle loading. Furthermore, **Figure 10e** demonstrates that the application of an external magnetic field significantly enhanced the magnetic retention of APS-MNP-loaded T cells under dynamic flow conditions. Increased nanoparticle loading and stronger magnetic gradients resulted in greater cellular displacement toward the magnetic source, confirming the feasibility of magnetic guidance for improving localization and persistence of adoptively transferred T cells (221).

Collectively, the findings summarized in **Figure 10** indicate that MNPs can simultaneously regulate T-cell receptor organization and facilitate magnetic targeting of therapeutic lymphocytes while preserving their cytotoxic and cytokine-producing functions, thereby providing a promising strategy for enhancing adoptive cancer immunotherapy.

## 6 Therapeutic performance and biosafety

The successful clinical translation of ROS-generating magnetic nanoplatforms depends not only on therapeutic potency but also on their safety profile, pharmacokinetics, and long-term biological behavior *in vivo*. Preclinical evaluation therefore requires a comprehensive assessment of both antitumor efficacy and potential adverse effects across multiple biological systems. Cellular studies provide essential information regarding catalytic activity, intracellular ROS generation, uptake efficiency, and mechanisms of cancer cell death induced by magnetic nanomaterials. Animal investigations further contribute to understanding biodistribution patterns, circulation time, tumor accumulation, clearance pathways, and therapeutic outcomes under physiologically relevant conditions. Particular attention has recently been directed toward evaluating potential toxicological effects associated with metal ion release, oxidative injury in healthy tissues, and long-term nanoparticle retention in major organs. Interactions with the immune system, hematological parameters, and hepatic or renal function have also become important considerations during safety assessment of multifunctional nanotherapeutics. Standardization of biological evaluation protocols is increasingly recognized as a prerequisite for comparing results across studies and facilitating regulatory approval processes. The following sections summarize the current approaches used to evaluate *in vitro* therapeutic performance, *in vivo* antitumor efficacy, and biosafety characteristics of MNPs designed for ROS-mediated cancer therapy.

### 6.1 *In vitro* and *in vivo* therapeutic evaluation

The biological performance of ROS-generating MNPs must be systematically evaluated through both *in vitro* and *in vivo* investigations to establish their therapeutic efficacy, mechanistic behavior, and translational potential. *In vitro* studies provide fundamental information regarding cellular interactions, intracellular trafficking, catalytic activity, and mechanisms of cell death, whereas *in vivo* experiments assess pharmacokinetics, biodistribution, tumor accumulation, therapeutic efficacy, and systemic safety under physiologically relevant conditions (222, 223).

Cellular uptake represents one of the earliest parameters examined during *in vitro* evaluation because insufficient internalization may severely limit catalytic activity regardless of nanoparticle composition (224). Uptake efficiency is commonly quantified using fluorescence microscopy, flow cytometry, inductively coupled plasma mass spectrometry (ICP-MS), and transmission electron microscopy to determine intracellular localization and nanoparticle distribution within subcellular compartments (225). Intracellular ROS generation is typically quantified using fluorescent probes such as dichlorodihydrofluorescein diacetate (DCFH-DA), hydroxyphenyl fluorescein (HPF), and singlet oxygen sensor green (SOSG), which enable visualization and quantification of oxidative species generated following nanoparticle treatment. These measurements provide direct evidence regarding catalytic activity and treatment responsiveness under biologically relevant conditions (226, 227).

Cytotoxicity is commonly assessed using metabolic assays including MTT, CCK-8, WST-1, and live/dead staining, allowing quantitative determination of dose-dependent therapeutic effects across different cancer cell lines. Comparative analysis using normal cells is particularly important for evaluating treatment selectivity and minimizing off-target toxicity (228). Mechanistic investigations frequently include measurements of mitochondrial membrane potential, ATP depletion, lipid peroxidation, glutathione depletion, caspase activation, and GPX4 expression in order to distinguish between apoptosis, ferroptosis, and other ROS-mediated death pathways. Such analyses provide insight into the molecular consequences of oxidative injury and facilitate mechanistic interpretation of therapeutic responses (229).

Although *in vitro* studies provide essential mechanistic information, they cannot accurately reproduce the complexity of vascular transport, immune interactions, protein corona formation, and tumor heterogeneity encountered in living organisms. Consequently, animal studies remain indispensable for evaluating the true therapeutic potential of magnetic nanoplatforms (177). Tumor-bearing murine models are the most frequently employed systems for evaluating antitumor efficacy because they allow quantitative assessment of tumor growth inhibition, survival outcomes, and treatment response over time. Parameters such as tumor volume reduction, inhibition rate, and progression-free survival are commonly used as indicators of therapeutic performance (18, 230).

Biodistribution studies are essential for determining nanoparticle accumulation in tumors and clearance from healthy organs. Techniques such as magnetic resonance imaging, fluorescence imaging, positron emission tomography, and ICP-MS analysis are frequently employed to quantify nanoparticle localization and organ-specific retention following systemic administration (231). Histopathological examination of tumor tissue provides additional evidence regarding treatment mechanisms by revealing necrosis, apoptosis, vascular damage, immune-cell infiltration, and oxidative injury after therapy. Simultaneous evaluation of liver, spleen, kidney, heart, and lung tissues is also necessary to identify potential systemic toxicity associated with nanoparticle exposure (232, 233). Recent investigations increasingly integrate imaging, therapy, and therapeutic monitoring into single theranostic platforms capable of simultaneously visualizing biodistribution and evaluating treatment efficacy in real time. Such multifunctional systems may significantly improve treatment personalization and facilitate clinical translation of ROS-mediated magnetic nanomedicine. As summarized in **Table 5**, representative magnetic nanoplatforms differ substantially in particle size and morphology, catalytic or functional efficiency, therapeutic modality, and level of *in vivo* validation. Direct comparison of these parameters provides a more informative framework for relating nanoplatform design to therapeutic performance and translational potential.

**Table 5:** Comparative physicochemical, catalytic, and therapeutic characteristics of representative ROS-generating magnetic nanoplatforms.

Magnetic nanoplatform	Nanoparticle size/shape	Catalytic/functional efficiency	Animal model	Therapeutic mode	Key outcomes	Ref.
Porous Fe_3_O_4_-GOx nanoparticles	Hollow porous spherical particles; ~200 nm diameter; ~50 nm shell thickness	GOx catalytic activity retained after encapsulation; efficient glucose consumption and H_2_O_2_/ROS generation; specific kinetic constant not reported	4T1 tumor-bearing BALB/c mice	GOx-mediated starvation therapy + PTT + MRI	Strong intracellular ROS generation and apoptosis; Fe_3_O_4_-GOx + NIR produced greater tumor suppression than either modality alone; negligible major-organ toxicity	(234)
CuS@Fe-MOF-DOX nanoplatform	Hexagonal CuS nanoplate core, ~85 nm; amorphous Fe-MOF shell, ~16 nm thick	Photothermal conversion efficiency: 39.7%; DOX loading efficiency: 27.5%; catalytic k-value not reported	Tumor-bearing mice	PTT + chemotherapy + MRI guidance	Stronger tumor inhibition than PTT or chemotherapy alone; effective MR/thermal imaging; no noticeable systemic adverse effects	(235)
Pd@CQD@Fe_3_O_4_ nanoparticles	Spherical; 63 ± 13 nm	ROS-generating activity demonstrated; specific catalytic k-value not reported	Not evaluated *in vivo*	ROS-mediated anticancer therapy/apoptosis induction	Total apoptosis: 79.8% in MCF-7 and 77.01% in SK-OV-3 cells; caspase-8 increased 1.40-fold in MCF-7 and 1.11-fold in SK-OV-3; caspase-3/7 increased 3.00-fold in SK-OV-3	(236)
Anti-PD-L1-functionalized Fe_3_O_4_@AuNR nanoplatform	Au nanorod core surrounded by Fe_3_O_4_ nanoclusters; overall particle size not explicitly reported in the summarized data	Stimuli-responsive drug release under acidic pH, elevated GSH, and NIR irradiation; specific catalytic k-value not reported	Ovarian tumor model in mice	Targeted chemotherapy + PTT + multimodal MRI/fluorescence imaging	Anti-PD-L1-mediated tumor targeting and dual-drug delivery; image-guided combination therapy improved antitumor efficacy	(237)
Fe_3_O_4_@ZnO/anti-EGFR/Nrf2-siRNA/brusatol nanoplatform	Fe_3_O_4_@ZnO nanoparticles <10 nm	Intracellular ROS increased by 191.09 ± 10.02%; specific kinetic constant not reported	Nude mice bearing cutaneous squamous cell carcinoma tumors	Magnetically targeted PDT + gene therapy + chemotherapy	Cell viability decreased by 80.43 ± 9.37%; *in vivo* tumor suppression reached 76.30 ± 5.12%	(238)
Fe_3_O_4_-MTX@HBc nanoparticles	Well-defined core-shell structure; hydrodynamic size 38.2 ± 5.6 nm	NIR-induced photothermal heating demonstrated; specific catalytic k-value not reported	4T1 tumor-bearing BALB/c mice	MRI-guided PTT + chemotherapy	Enhanced tumor accumulation and strong chemo-photothermal tumor inhibition; no obvious major-organ damage during treatment	(239)

Overall, the combined use of *in vitro* mechanistic studies and *in vivo* therapeutic investigations provides a comprehensive framework for evaluating magnetic nanoparticle performance and identifying the most promising candidates for future clinical applications.

### 6.2 Biocompatibility and toxicological considerations

The clinical translation of ROS-generating MNPs depends not only on therapeutic efficacy but also on their ability to maintain an acceptable safety profile following repeated or long-term exposure. Excessive oxidative activity that is beneficial inside tumors may become harmful if catalytic reactions occur in healthy tissues, making biosafety evaluation a central component of nanomedicine development (25, 240).

Particle size, morphology, composition, and surface chemistry strongly influence biological compatibility by regulating circulation behavior, cellular interactions, and organ accumulation after systemic administration. Small variations in these physicochemical properties can significantly alter toxicity outcomes even among nanoparticles sharing the same magnetic core composition (135, 241). Following intravenous injection, MNPs frequently accumulate within the liver and spleen because these organs are major components of the mononuclear phagocyte system responsible for nanoparticle clearance. Consequently, hepatic and splenic toxicity are among the most closely monitored endpoints during preclinical evaluation (240, 242). One of the major safety concerns associated with iron-containing nanomaterials is uncontrolled iron release, which may induce oxidative damage through excessive ROS production and disruption of intracellular antioxidant defenses. Such effects can contribute to mitochondrial dysfunction, membrane injury, and inflammatory responses in non-target tissues (243).

Surface engineering has emerged as one of the most effective approaches for reducing toxicity because biocompatible coatings can improve colloidal stability and reduce nonspecific cellular interactions. Polyethylene glycol, polysaccharides, and lipid coatings are widely used to minimize aggregation and improve biological tolerance (25, 244). The formation of a protein corona after exposure to biological fluids represents another important determinant of nanoparticle safety because adsorbed proteins can modify cellular uptake, biodistribution, and immune recognition. Recent evidence suggests that the protein corona may exert a greater influence on *in vivo* fate than core size or hydrodynamic diameter alone (245).

Immunotoxicity has also become an important area of investigation because nanoparticle exposure can activate inflammatory pathways, alter macrophage function, and influence cytokine secretion profiles. Careful regulation of immune interactions is therefore essential for maintaining therapeutic selectivity and minimizing adverse responses (246). Comprehensive biosafety studies typically include hematological analysis together with liver and kidney function tests to identify potential systemic toxicity following treatment. Histological examination of major organs remains essential for detecting tissue damage that may not be apparent through biochemical markers alone (135, 240). Long-term retention and biodegradation behavior are additional considerations because persistent nanoparticle accumulation may increase the risk of delayed toxicity after treatment completion. Understanding degradation pathways and clearance mechanisms is therefore critical for future clinical applications (247).

Long-term clearance of MNPs is strongly size- and formulation-dependent, and hydrodynamic diameter is more informative than the inorganic core diameter alone. Ultrasmall iron oxide particles with hydrodynamic dimensions near or below the glomerular filtration threshold can undergo rapid renal elimination. For example, USPIO formulations with a hydrodynamic diameter of approximately 4.7 nm showed about 65% renal clearance within 4 h, whereas another formulation with a hydrodynamic size of approximately 5 nm achieved about 92% urinary excretion within 8 h and nearly complete clearance within 24 h (248). These findings also indicate that renal clearance should not be predicted solely from core size, because surface coating, charge, protein adsorption, and aggregation can increase the effective hydrodynamic diameter and redirect nanoparticles toward the mononuclear phagocyte system.

Larger assemblies that exceed the renal filtration threshold are more commonly retained by the liver and spleen and cleared through slower hepatobiliary, metabolic, or macrophage-mediated pathways. This distinction is illustrated by micelle-encapsulated extremely small iron oxide nanoparticles with an average hydrodynamic size of 9.35 nm. Despite their small inorganic cores, renal excretion accounted for only about 4.45% of the injected dose at 24 h, whereas nearly 40% was eliminated through the hepatobiliary route within the same period (249). Long-term retention can be considerably longer when MNPs are internalized by tissue macrophages. In a 15-month study of 12-nm iron oxide cores with different coatings, magnetic particle signals disappeared from the liver within several months, whereas residual particles remained detectable in the spleen even at 15 months. The same study showed progressive conversion of nanoparticle-derived iron into ferritin-associated iron pools (250). Therefore, degraded MNP components should not be regarded as being immediately excreted. Released Fe²^+^ and Fe³^+^ can enter endogenous ferritin, transferrin, hemoglobin, and other physiological iron pathways, while intact particles, coatings, and soluble degradation products may follow different renal or hepatobiliary clearance routes.

Representative biosafety studies have demonstrated that the biological fate and toxicity of magnetic nanoplatforms strongly depend on particle composition, surface modification, administration route, and dosage. These size-dependent clearance pathways should be interpreted together with systemic toxicity endpoints. As summarized in **Table 6**, representative MNP formulations show marked differences in organ retention and clearance behavior, while hepatic and renal safety is commonly assessed through ALT, AST, BUN, and creatinine measurements across different doses and follow-up periods.

**Table 6:** Comparative biodistribution, clearance, and systemic toxicity profiles of representative magnetic nanoplatforms.

Magnetic nanoplatform	Dose/follow-up	Biodistribution/clearance	ALT/AST	BUN/creatinine	Histopathological/systemic findings	Ref.
Bio-Fe_3_O_4_ nanoparticles	100 mg kg^-^¹	No abnormal accumulation pattern reported	NR	NR	CBC unchanged; no platelet aggregation; no abnormalities in liver, spleen, or kidney	(251)
Salicylic acid-functionalized Fe_3_O_4_ nanoparticles (SaIONPs)	IV or IG administration	IV administration promoted uptake by periportal Kupffer cells and hepatocytes, whereas IG administration mainly resulted in hepatocyte accumulation	NR	NR	Faster recovery of hemoglobin levels; no major hepatic injury reported	(252)
PEG-coated Fe_3_O_4_ nanoparticles	Up to 90 mg kg^-^¹; 14 days	Spleen was a major elimination organ; nanoparticles accumulated predominantly in macrophage lysosomes	Serum biochemical changes observed only at the highest dose; individual ALT/AST values NR	Individual BUN/creatinine values NR	Mild toxicity at 90 mg kg^-^¹; increased organ coefficients in spleen, liver, heart, and kidney	(253)
Fe_2_O_3_ nanoparticles	150 and 300 mg kg^-^¹	Liver: ~80–90%; spleen: 5–8%; bone marrow: 1–2%	ALT and AST increased at 150 and 300 mg kg^-^¹; ALP also increased	NR	WBC increased at medium/high doses; severe vascular congestion, sinusoidal dilation, and erythrocyte aggregation at 300 mg kg^-^¹	(254)
Fe_3_O_4_-loaded ultrasound nanodroplets (MPFS@NDs)	Therapeutic study	Tumor-targeted accumulation through macrophage-targeting strategy	No obvious hepatic toxicity reported	No obvious renal toxicity reported	No significant pathological damage in major organs	(252)
PAA-coated IONPs	2.5, 10, and 20 mg kg^-^¹; up to 28 days	Predominant uptake by RES organs, particularly liver and spleen	ALT remained within the normal range. Day 1: 17.18 ± 2.17, 17.58 ± 1.31, and 16.29 ± 2.07 U L^-^¹ at 2.5, 10, and 20 mg kg^-^¹, respectively; AST showed no significant increase	BUN and creatinine remained within normal ranges without significant differences from control	No obvious histopathological injury in major organs during the 28-day observation period	(255)
SFN/BQR1@MSPION3	Fe dose: 5 or 15 mg kg^-^¹	*In vivo* tumor-bearing mouse evaluation	ALT and AST showed no significant difference from control	BUN and creatinine showed no significant difference from control	No discernible adverse effect on liver, kidney, or heart function even at 15 mg Fe kg^-^¹	(256)
PEG-BP and BNF-PAA iron oxide nanoparticles	Single IV exposure; 60-day follow-up	Coating-dependent distribution; major accumulation in liver and spleen, with additional formulation-dependent kidney and tumor uptake	ALT and AST remained within normal ranges at day 60	Creatinine remained within normal range; BNF-PAA caused a statistically significant increase in BUN, but the value remained within the physiological range	No consistent organ toxicity; early immune-cell alterations normalized by day 60	(257)

Recent advances in activatable and stimuli-responsive magnetic nanoplatforms aim to improve safety by restricting catalytic activity to the tumor microenvironment and minimizing exposure of healthy tissues to oxidative stress. Such strategies are expected to improve the therapeutic index of ROS-mediated nanomedicine and accelerate regulatory approval in the future.

### 6.3 Protein corona formation and long-term biotransformation of MNPs

Once MNPs enter blood or other protein-rich biological fluids, their synthetic surface identity is rapidly replaced by a biomolecular interface formed through adsorption of plasma proteins. This protein corona is dynamic and depends on nanoparticle size, surface chemistry, coating composition, and the surrounding biological medium. Its formation can partially shield targeting ligands and modify receptor accessibility, cellular uptake, and interactions with the mononuclear phagocyte system. Experimental studies with functionalized MNPs have shown that nonspecific serum protein adsorption can change target affinity by masking or competing with surface recognition sites (258). The composition of the corona is also strongly coating-dependent. For example, citrate- and riboflavin-coated SPIONs formed distinct serum-protein profiles despite relatively similar physicochemical characteristics, and these differences were associated with altered cellular uptake in breast cancer cells (259). Therefore, magnetic targeting *in vivo* should not be interpreted solely from the magnetic properties measured before administration. Even when the magnetic core remains functional, corona-induced changes in hydrodynamic size, colloidal state, ligand exposure, macrophage recognition, and circulation time can alter the effective transport and retention of MNPs under an applied magnetic field.

The biological identity of MNPs continues to evolve after cellular internalization. Following endocytosis, iron oxide-based MNPs are commonly trafficked through endosomal compartments and subsequently exposed to the acidic and chemically complex lysosomal environment. Low pH favors progressive dissolution of the iron oxide lattice, while proteins, reducing agents, and chelating molecules can either accelerate or delay this process. Recent experimental work showed that acidic conditions markedly accelerate IONP degradation, whereas albumin can stabilize the particles and reduce dissolution. In contrast, glutathione accelerates degradation, and citrate produces particularly rapid dissolution under biologically relevant conditions (260). Released Fe²^+^ and Fe³^+^ ions can enter the cellular labile iron pool and subsequently be incorporated into normal iron-metabolism pathways. A substantial fraction can be sequestered by ferritin, while iron may also be redirected toward heme or iron-sulfur cluster synthesis. Over longer periods, this process can transform initially crystalline and strongly magnetic iron oxide cores into partially dissolved or weakly magnetic iron-containing species. The extent and kinetics of this transformation depend strongly on coating stability, particle architecture, intracellular localization, dose, and residence time. Also, incomplete degradation may permit persistent lysosomal retention, whereas progressive dissolution can alter both magnetic responsiveness and catalytic ROS-generating capacity. Thus, long-term biological performance is determined by a changing continuum from the administered nanoplatform to corona-coated particles, lysosomal degradation intermediates, released iron ions, and endogenous iron-storage products such as ferritin (261).

## 7 Current challenges and future perspectives

Despite remarkable advances in ROS-generating magnetic nanoplatforms for cancer therapy, several scientific and translational challenges continue to limit their clinical implementation. One of the major limitations of current systems is the strong dependence of catalytic performance on tumor-specific biochemical conditions, including hydrogen peroxide concentration, acidity, oxygen availability, and antioxidant capacity, which may vary considerably among tumor types and even within different regions of the same tumor, resulting in heterogeneous therapeutic responses (43, 262). Furthermore, insufficient catalytic efficiency under physiological conditions, premature nanoparticle clearance, limited tumor penetration, and off-target accumulation remain significant barriers to achieving consistent therapeutic outcomes (11, 263). The complex interplay between particle size, surface chemistry, composition, and biodegradation behavior further complicates the optimization of magnetic nanoplatform design and may lead to unpredictable biological interactions following systemic administration (160, 264).

Clinical translation presents additional obstacles beyond therapeutic performance alone. Large-scale and reproducible manufacturing of multifunctional MNPs remains technically challenging due to difficulties in maintaining batch-to-batch consistency in size distribution, surface modification, and catalytic activity. Regulatory approval is further complicated by the absence of standardized protocols for evaluating biodistribution, long-term toxicity, biodegradation kinetics, immunological responses, and environmental safety (265–267). In addition, many preclinical studies rely heavily on simplified murine tumor models that fail to accurately reproduce the complexity, heterogeneity, and immune characteristics of human cancers, limiting the predictive value of current efficacy data (267). Economic considerations, manufacturing costs, and scalability issues also represent important factors that may influence future commercialization and clinical adoption of these technologies (264).

Future research is expected to focus on the development of highly intelligent magnetic nanoplatforms capable of dynamically adapting to the biological characteristics of individual tumors. Multi-stimuli-responsive systems, programmable catalytic nanomaterials, and self-regulating ROS generators may provide improved control over therapeutic activation while minimizing unwanted toxicity in healthy tissues (160, 268). Advances in artificial intelligence, computational materials design, and machine learning-assisted nanomedicine are also expected to accelerate the discovery of optimized nanoparticle architectures with improved therapeutic indices (269, 270). Moreover, integration of catalytic therapy with immunotherapy, gene therapy, and precision oncology approaches may enable highly personalized treatment strategies tailored to patient-specific molecular profiles (267). The emergence of clinically translatable theranostic nanoplatforms combining diagnosis, treatment, and real-time therapeutic monitoring within a single system is likely to become a major direction in next-generation cancer nanomedicine (264, 271). In addition to therapeutic optimization, future magnetic nanoplatforms are expected to incorporate real-time diagnostic and monitoring capabilities. Recent developments in smart nanobiosensors, including MXene quantum dot-based surface plasmon resonance systems, demonstrate how advanced nanomaterials can achieve highly sensitive biomedical detection while supporting the broader evolution of integrated theranostic platforms. The combination of responsive nanomaterials with biosensing technologies may facilitate personalized treatment monitoring and precision nanomedicine in future cancer management (272). Ultimately, overcoming current biological, technological, and regulatory challenges will be essential for transforming magnetic ROS nanoplatforms from promising laboratory concepts into clinically viable therapeutic technologies for precision cancer treatment.

## 8 Conclusions

The rapid development of ROS-generating magnetic nanoplatforms has fundamentally expanded the role of MNPs from passive carriers and imaging agents to active therapeutic mediators capable of reshaping tumor redox homeostasis. The major scientific significance of this field lies in its ability to exploit intrinsic biochemical vulnerabilities of cancer cells, particularly their dependence on fragile oxidative balance and altered metabolic environments, to achieve selective therapeutic intervention. Rather than functioning through a single mechanism, magnetic ROS nanotherapeutics operate through interconnected pathways involving catalytic oxidation, lipid peroxidation, mitochondrial dysfunction, and immune modulation, highlighting their potential to address the multifactorial nature of tumor progression and therapeutic resistance.

Nevertheless, the impressive performance reported in experimental studies has not yet translated into comparable clinical success. A major gap remains between simplified laboratory models and the biological complexity encountered in human malignancies. Tumor heterogeneity, variable redox conditions, dynamic immune interactions, and patient-to-patient variability can substantially influence catalytic performance and therapeutic outcomes. In addition, many currently available nanoplatforms rely on increasingly sophisticated multifunctional architectures whose complexity may hinder reproducibility, large-scale production, and regulatory approval.

Another unresolved challenge concerns the establishment of standardized evaluation criteria for therapeutic efficacy, biodegradation, long-term biosafety, and immunological compatibility. The lack of harmonized testing protocols makes direct comparison between studies difficult and slows the identification of clinically relevant design principles. Furthermore, insufficient understanding of nanoparticle fate after treatment, particularly regarding long-term retention and metabolic clearance, continues to represent an important barrier to clinical translation.

Future progress in this field will likely depend less on increasing structural complexity and more on improving biological adaptability and translational feasibility. The next generation of magnetic nanoplatforms is expected to incorporate intelligent responsiveness, programmable catalytic behavior, and personalized therapeutic capabilities tailored to the biochemical characteristics of individual tumors. Integration with artificial intelligence-assisted materials discovery, computational modeling, and precision oncology frameworks may accelerate the identification of clinically optimal nanotherapeutic systems.

Ultimately, the future impact of magnetic ROS nanomedicine will depend on its ability to bridge the gap between catalytic innovation and clinical practicality. Achieving this transition will require close collaboration among materials scientists, engineers, oncologists, immunologists, and regulatory experts. If these challenges can be successfully addressed, ROS-generating MNPs may evolve from promising experimental technologies into a realistic component of precision cancer therapy in the coming decades.

## Glossary

ACSL4: Acyl-CoA synthetase long-chain family member 4
ALP: Alkaline phosphatase
ALT: Alanine aminotransferase
Apaf-1: Apoptotic protease-activating factor 1
AST: Aspartate aminotransferase
ATP: Adenosine triphosphate
BAK: Bcl-2 homologous antagonist/killer
BAX: Bcl-2-associated X protein
BET: Brunauer–Emmett–Teller
BUN: Blood urea nitrogen
CAT: Catalase
CCK-8: Cell Counting Kit-8
CDT: Chemodynamic therapy
CTLA-4: Cytotoxic T-lymphocyte-associated protein 4
DAMPs: Danger-associated molecular patterns
DCFH-DA: 2′,7′-Dichlorodihydrofluorescein diacetate
DOX: Doxorubicin
EPR: Enhanced permeability and retention
FESEM: Field-emission scanning electron microscopy
FITC: Fluorescein isothiocyanate
GOx: Glucose oxidase
GPX4: Glutathione peroxidase 4
GSH: Reduced glutathione
GSSG: Oxidized glutathione
HGB: Hemoglobin
HMGB1: High-mobility group box 1
HPF: Hydroxyphenyl fluorescein
ICD: Immunogenic cell death
ICP-MS: Inductively coupled plasma mass spectrometry
IONPs: Iron oxide nanoparticles
MHT: Magnetic hyperthermia
MNPs: Magnetic nanoparticles
MOMP: Mitochondrial outer membrane permeabilization
MOF: Metal-organic framework
MRI: Magnetic resonance imaging
MTT: 3-(4,5-Dimethylthiazol-2-yl)-2,5-diphenyltetrazolium bromide
NIR: Near-infrared
PAA: Poly(acrylic acid)
PAL: Phenylalanine ammonia-lyase
PBS: Phosphate-buffered saline
PD-1: Programmed cell death protein 1
PD-L1: Programmed death-ligand 1
PDT: Photodynamic therapy
PEG: Polyethylene glycol
PLGA: Poly(lactic-co-glycolic acid)
POX: Peroxidase
PTT: Photothermal therapy
ROS: Reactive oxygen species
SLC7A11: Solute carrier family 7 member 11
SOD: Superoxide dismutase
SOSG: Singlet oxygen sensor green
SPIONs: Superparamagnetic iron oxide nanoparticles
TEM: Transmission electron microscopy
TGA: Thermogravimetric analysis
TMB: 3,3′,5,5′-Tetramethylbenzidine
TME: Tumor microenvironment
USIO NCs: Ultrasmall iron oxide nanoclusters
WBC: White blood cell count
WST-1: Water-soluble tetrazolium salt-1
XRD: X-ray diffraction
